# 4D live imaging and computational modeling of a functional gut-on-a-chip evaluate how peristalsis facilitates enteric pathogen invasion

**DOI:** 10.1126/sciadv.abo5767

**Published:** 2022-10-21

**Authors:** Aleix Boquet-Pujadas, Thomas Feaugas, Alba Petracchini, Alexandre Grassart, Héloïse Mary, Maria Manich, Samy Gobaa, Jean-Christophe Olivo-Marin, Nathalie Sauvonnet, Elisabeth Labruyère

**Affiliations:** ^1^ Bioimage Analysis Unit, Institut Pasteur, Université Paris Cité, Paris, France.; ^2^ Intracellular Trafficking and Tissue Homeostasis Group, Institut Pasteur, Université Paris Cité, Paris, France.; ^3^ Biomedical Imaging Group, École polytechnique fédérale de Lausanne (EPFL), Lausanne, Switzerland.; ^4^ Intracellular Trafficking and Tissue Homeostasis Group, Institut Pasteur, Paris, France.; ^5^ Unit of Bioengineering and Microbiology, Center for Microbes, Development and Health (CMDH), Institut Pasteur of Shanghai, Chinese Academy of Sciences, Shanghai, China.; ^6^ Biomaterials and Microfluidics Core Facility, Institut Pasteur, Université Paris Cité, Paris, France.

## Abstract

Physical forces are essential to biological function, but their impact at the tissue level is not fully understood. The gut is under continuous mechanical stress because of peristalsis. To assess the influence of mechanical cues on enteropathogen invasion, we combine computational imaging with a mechanically active gut-on-a-chip. After infecting the device with either of two microbes, we image their behavior in real time while mapping the mechanical stress within the tissue. This is achieved by reconstructing three-dimensional videos of the ongoing invasion and leveraging on-manifold inverse problems together with viscoelastic rheology. Our results show that peristalsis accelerates the destruction and invasion of intestinal tissue by *Entamoeba histolytica* and colonization by *Shigella flexneri*. Local tension facilitates parasite penetration and activates virulence genes in the bacteria. Overall, our work highlights the fundamental role of physical cues during host-pathogen interactions and introduces a framework that opens the door to study mechanobiology on deformable tissues.

## Teaser

Image-based measurements in a deforming colon-like environment show that local tissue tension helps human enteropathogens invade.

## INTRODUCTION

The fundamental importance of environmental stimuli to cell behavior has sparked a quest for evermore physiologically relevant experiments. Cells integrate information stemming from multicellular interactions, as well as external biochemical and physical cues. For example, the human pathogen *Entamoeba histolytica* interacts closely with the epithelium and regulates its migration strategy according to the density of the extracellular matrix (ECM) (*1*), as well as to chemoattractants such as proinflammatory cytokines (*2*). On the other hand, mechanical cues such as shear force modulate gene expression in *Salmonella* while inducing differentiation in the host (*3*, *4*). Shear stress resulting from fluid flow has also been shown to promote bacterial adhesion in the urinary tract (*5*). More generally, the development of force probes at the microscale has recently helped elucidate the impact of physical interactions on tissue morphogenesis and cell function (*6*), including tasks as diverse as migration, metabolic regulation (*7*, *8*), and transcription (*9*–*11*).

To reproduce the subset of external stimuli relevant to the pathology under study without the complications of grafts, explants, or in vivo experiments, recent efforts have focused on engineering new microenvironments in vitro. The cell culture platforms resulting from this endeavor are many and range from bioreactor suspensions where cells self-organize (*12*) to three-dimensional (3D) ECM scaffolds where progenitors cells embed to differentiate and form organoids (*13*) and, most recently, to microfabricated chips that harness microfluidics to mimic different organ functions and introduce mechanical stress (*14*).

In this context, organs-on-chip (OOCs) do not only enable the interaction between different cell types but also feature a porous membrane that can maintain distinct apical and basal conditions, thereby promoting tissue homeostasis, impermeability, and functional polarization (*15*). In addition, OOCs can typically recapitulate nutrient flow and mechanical peristalsis through microfluidic design. The combination of all these factors results in dynamic tissue-like structures that are relevant to many research directions, notably host-pathogen interactions. For instance, it has been shown that the topology and peristaltic cycle simulated by guts-on-chip (*16*) are essential to reproduce the virulent outcome of *Shigella flexneri* infections (*17*) reported in clinical studies (*18*), which could not otherwise be observed in the face of simpler epithelial monolayers of colon cell lines (*19*) or even of enteroids from human biopsies (*20*, *21*).

To observe the process under study, OOCs are typically fixed at a time point of interest and cut into thin slices that can later be imaged. Despite their success at reproducing some of the key characteristics of in vivo physiology, the study of dynamical phenomena in OOCs is complicated by multiple imaging challenges (*22*), especially in deformable OOCs such as intestine-chips (*23*). The motion and curvature of the tissue caused by the deformation prevent proper 2D confocal slicing, whereas 3D acquisitions cannot keep pace with the moving “organ.” Another complication arises from the architecture of some OOCs: Often, the multiple channels compound with the thickness of the polydimethylsiloxane (PDMS) layer at the bottom to require a working distance that is challenging to live microscopy. To this day, these obstacles have only been partially circumvented by the use of a few in-house light sheet microscopes that integrate chips directly into their optical design, for example, through optical fiber (*24*, *25*). As a consequence, OOCs are nearly always studied as fixed samples. Another burden to OOCs is the difficulty of probing cellular mechanics because the chip case renders the tissue inaccessible. Together, all these technical issues hinder real-time studies of transient events in these more physiologically relevant models and stall any mechanobiological studies thereof against current research interests (*26*).

This is also the case for host-pathogen interactions, which have been studied almost exclusively in fixed long-term experiments. In the gut, for example, *S. flexneri* makes its way into the intestinal barrier by penetrating epithelial cells via a type III secretion system (T3SS), which injects a number of effector proteins that eventually trigger its own internalization (*27*). Once inside, the bacterium exits its vacuole and hijacks additional mechanisms to replicate and spread to neighboring cells, eventually degrading the enteric barrier at the tissue scale. To initially reach its host cell, however, other than the T3SS itself, *S. flexneri* lacks robust adhesive and motion devices. Intestine-chips were key in showing how the bacteria profit instead from the topology of the tissue, avoiding the use of adhesins that could otherwise make them conspicuous to the immune system; they also showed that the peristaltic movement is essential to boost the effectivity of the infection (*17*). Nevertheless, the dynamical processes and mechanical interactions behind this efficient infection process remain unknown for lack of ways to reliably follow the temporal evolution of pathogens as they physicochemically interact with the tissue, notably at the early stages of infection (*28*).

Other virulent enteropathogens have yet to be studied in OOCs despite the demonstrated influence of the intestinal environment on their behavior (*29*). For example, *E. histolytica*, the causative agent of amebiasis (*30*), also invades the polarized epithelial barrier of the gut, although its strategy is different from that of *Shigella*: The parasite relies on phagocytosis and pericellular proteolysis instead (*31*, *32*). Both these mechanisms are bolstered by chemical interactions with the host cells. The 3D architecture of the tissue is also key to the parasite’s motility (*33*), similarly highlighting the importance of physical considerations (*34*). By combining transient adhesions regulated by the Gal/GalNac lectin with myosin-based blebbing activity (*35*), *E. histolytica* migrates in typical amoeboid fashion. Precisely owing to their high motility, amoebae are especially unsuited to fixed studies. Therefore, the role of mechanical peristalsis in their pathogenicity, as well as their dynamic interactions with the gut, have remained nearly uncharted.

To study dynamic host-pathogen interactions in physiologically relevant contexts, we propose a framework that enables 4D live imaging of OOCs under cyclic deformations such as intestine-chips. This approach also allows probing the rheology of the tissue and estimating the spatiotemporal distribution of mechanical stress within the tissue in real time. The resulting imaging framework achieves the temporal resolution required for real-time studies of host-pathogen interactions and effectively bypasses the physical inaccessibility of the chips, originally complicated by their encasing. In this way, we broaden the scope of OOCs and open the floor to mechanobiological inquiries. We validate our framework on intestine-chips, which reproduce the enteric barrier under peristalsis and thus constitute a first-class platform to explore the role of physical cues in host-pathogen interactions. We study the infection dynamics associated with *E. histolytica* and *S. flexneri* to find that both microbes benefit from peristalsis, albeit differently. To this end, we show that different quantitative measures of invasion such as cell death or faster activation of virulent genes, respectively, correlate with tissue stress at a local level. This demonstrates that the two human pathogens adapt to the colonic environment by exploiting mechanical forces in different ways. For example, membrane or junction tension induces virulence in *S. flexneri*, accelerating cell-to-cell spreading, whereas physical movement increases their colonization speed. In another example, peristalsis enhances the tissue destruction caused by the lysing activity of *E. histolytica* while helping the parasite penetrate faster through zones of higher tension. Together, these results bring new insight into the role of mechanical cues in general—and of gut peristalsis in particular—during the early stages of infection and attest to the versatility of our framework for analyzing the dynamics of host-pathogen interactions in situ using OOCs.

## RESULTS

**Fig. 1.**
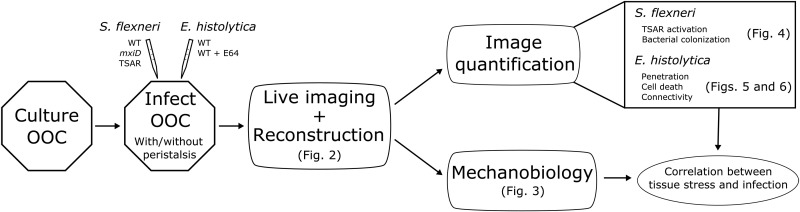
Schematic of the end-to-end workflow. Diagram including the full workflow in relation with the other figures. Find full description in Image processing and mechanobiology for deformable OOCs. WT, wild type.

To mimic the gut, we used intestine-chips (Emulate; see Materials and Methods) matured with either the human colonic cell line Caco2 or its CRISPR-Cas9 derivative edited to express E-cadherin–green fluorescent protein (GFP). We applied a cyclic stretch of magnitude of 10% and frequency of 0.15 Hz (~*T* = 6.6-s period) to the chips to reproduce the peristalsis of the colon. These values were chosen according to clinical studies and coincide with those found most physiological in the culture of intestine-chips (*16*, *36*, *37*). In the “Image processing and mechanobiology for deformable OOCs” section, we first describe a novel methodology to image these intestine-chip systems live and continue by introducing a framework to conduct mechanobiological research thereon. In the “Revealing the dynamics of pathogen invasion using image processing and mechanobiology” section, we leverage the resulting framework to study the invasion of enteric pathogens in a quantitative manner. A visual summary of the end-to-end workflow is provided in Fig. 1.

### Image processing and mechanobiology for deformable OOCs

**Fig. 2.**
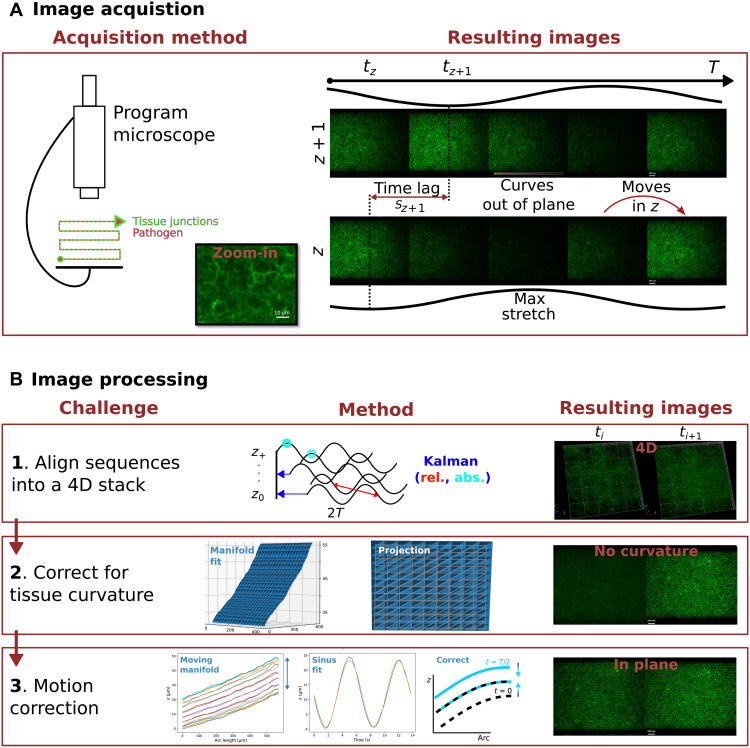
Reconstruction and visualization of deformable OOCs. (**A**) Image acquisition. The microscope is programmed to take a video sequence for twice the peristaltic period at each *z* position and for each channel. A zoom-in shows the E-cadherin structures that make up the tissue signal. The right part of the figure shows a single period for two sequences (that are) consecutive in *z*. The resulting sequences pose three challenges. First, they are out of phase: There is a time lag *s*_*z*+1_ between two *z*-consecutive sequences (notice that the bottom sequence lags behind the top one). Second, the tissue is not flat: The curvature takes a part thereof out of focus even within a same image (notice that half the image is dark). Third, the tissue disappears cyclically, moving out of focus with the stretch (it “leaves” the image and comes back for the same *z*). (**B**) Image processing. To fix these issues, we propose three consecutive processing steps; we illustrate them with two consecutive images: *t_i_* and *t*_*i*+1_. (1) We use a Kalman filter to align all cyclic videos in time. This is done for all *z*, resulting in a 4D reconstruction of the deforming tissue. (2) To flatten the curved tissue, a manifold is fitted and projected onto 2D (bringing the tissue in focus). (3) The out-of-plane movement caused by the tissue moving is corrected with a fitted sinusoid. The remaining movement is the stretching of the tissue (notice that it stays visible in both images).

**Fig. 3.**
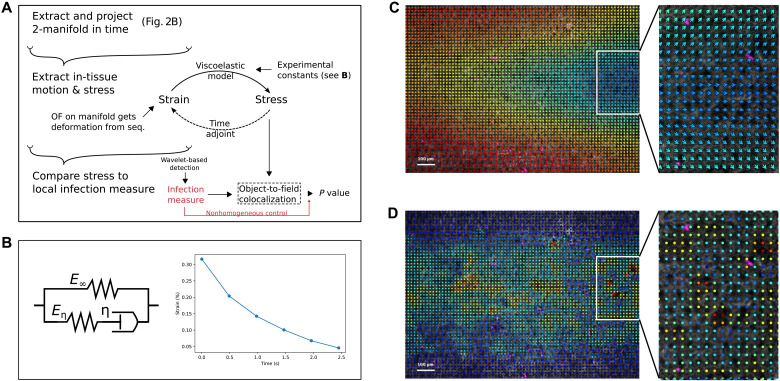
Rheological characterization of the tissue and computation of spatiotemporal stress maps and statistical colocalizations thereof. (**A**) Schematic of the process from the reconstruction of the moving tissue to the computation of stress on the manifold projection and to the colocalization analysis with other measures. (**B**) Schematic: Standard linear solid (SLS) model describing the response of a viscoelastic material through a combination of springs and dashpots. Right: Creeping response of the strain toward equilibrium (purely elastic would be instantaneous) once the stress is removed. The decaying exponential is governed by the retardation time τ_c_. (**C**) Displacement map of the tissue obtained from manifold projection (arrows mark direction, blue low to high red; scale bar, 100 μm) at maximum pressure shows how different parts of the tissue are stretched differently. Pathogens are in violet and tissue in white. (**D**) Stress map of the tissue obtained from (C) shows the spatial heterogeneity of the tension across different junctions.

**Fig. 4.**
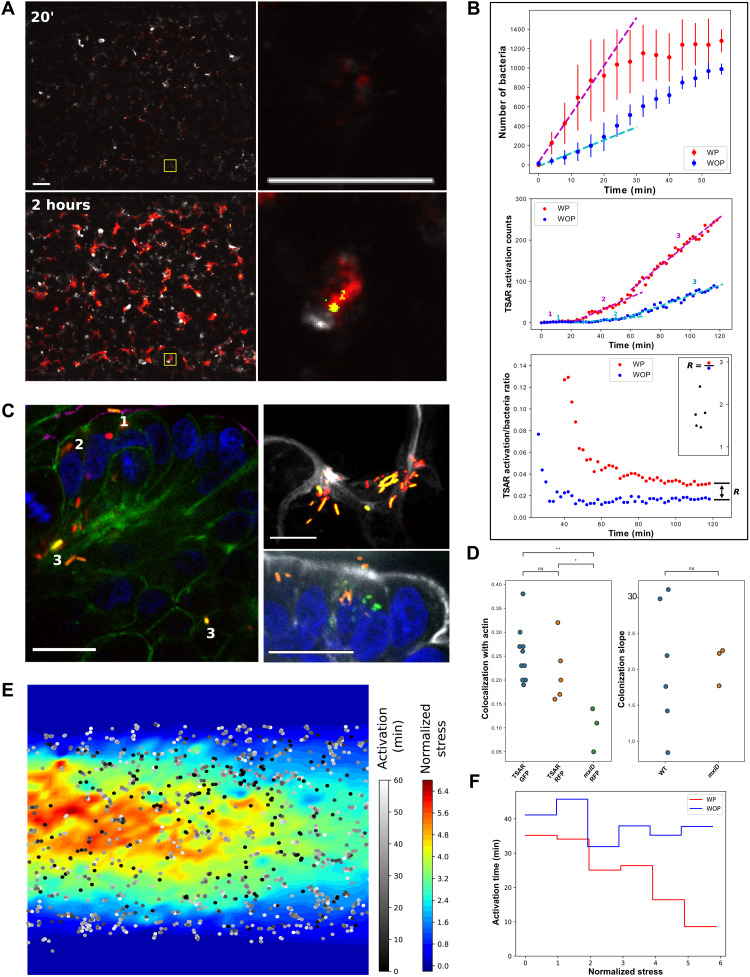
Quantitative dynamics of tissue invasion by *S. flexneri*. (**A**) Live spinning-disk confocal images of OOCs after 20 min or 2 hours of colonization with *S. flexneri* (TSAR strain) expressing red fluorescent protein (RFP; red) constitutively and GFP (green) upon T3SS activation and labeled in live for actin with SiR-Actin (white). Scale bars, 200 μm and 20 μm for zoom-ins. (**B**) Quantitative measures of the phenomena in (A) over time, WP-WOP comparisons. Top: Number of bacteria in the growing colonies. The lines show (least-squares) linear regressions of the onset. *n* = 8 chips. Middle: Example curves of the number of TSAR-activated bacteria as a marker of their secretion system. Three distinct phases of invasion are singled out with a linear fit. *n* = 4 chips. Bottom: Example curves of the ratio of activated bacteria to the total number of bacteria. Inset: Ratio between WP and WOP ratios at the plateau. *n* = 5 chips. (**C**) Fixed images of *S. flexneri* interacting with OOCs during different phases of the infection: Bacterial entry and cell-to-cell spread displaying, or not, T3SS activation (labels 1 to 3). (**D**) Left: Statistical colocalization of actin with TSAR *Shigella* expressing GFP and RFP and with the *mxiD* strain. Right: Ratio between the WP-WOP colonization slopes in (A) and (B) compared to the ratio for the nonvirulent *mxiD* strain. Unequal-variances *t* test. (**E**) Stress map (averaged over a cycle) with TSAR activation times superimposed; every point corresponds to the (*x*, *y*) coordinates of a tracked activation. (**F**) Histogramic relation showing the correlation between stress and TSAR activation time WP compared to a WOP proxy.

**Fig. 5.**
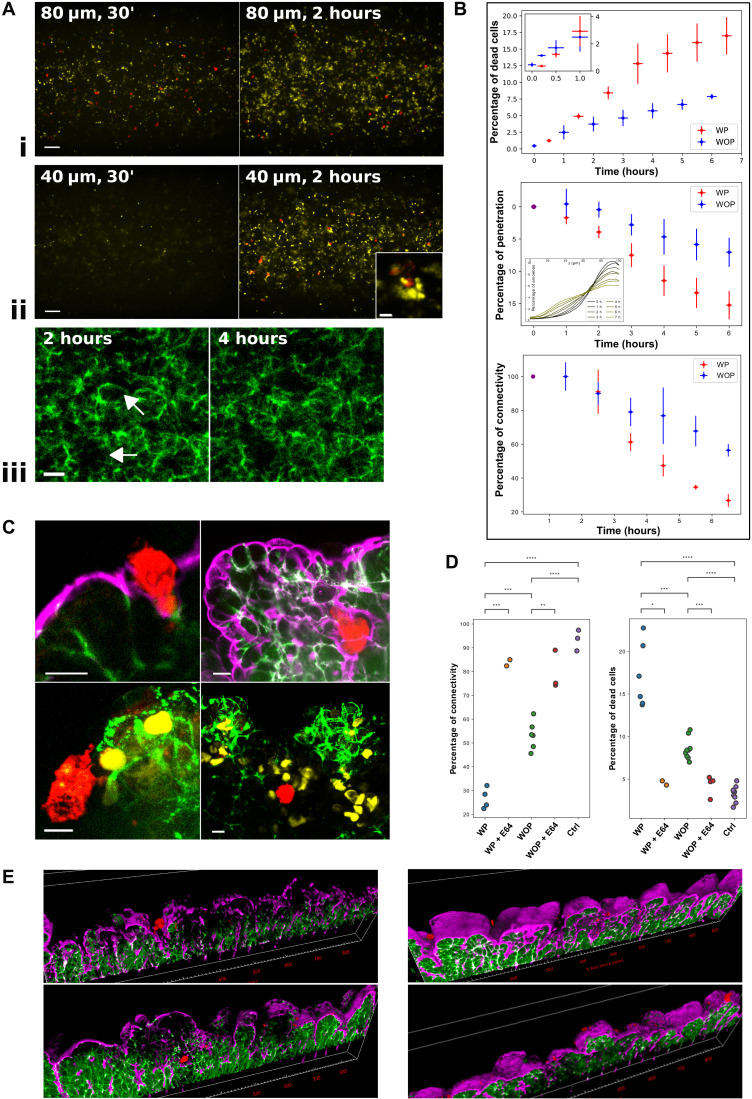
Quantitative dynamics of tissue invasion by *E. histolytica*. (**A**) Live spinning-disk confocal images of OOCs. Amoebae are labeled with red cell tracker (red), dead cells with Draq7 (yellow), and actin with SiR-Actin (violet), and Caco2 cells express E-cadherin (green). (i and ii) Amoebae kill epithelial cells and penetrate deeper over time (30 min, 2 hours). Toward the top (80 μm from the basal side) and middle (40 μm) of the tissue. Scale bars, 200 μm. Inset: Trophozoite that penetrated under the epithelium, killing the surrounding tissue cells. Scale bar, 20 μm. (iii) Tight junctions are destroyed over time; arrows point at parts of junctions that were cut. Scale bar, 20 μm. (**B**) Quantitative measures of the phenomena in (A) over time, WP-WOP comparisons. Top: Percentage of dead cells to total cells. Destruction WP quickly overtakes that of WOP. Inset: Close-up at 1 hour. *n* = 8 chips. Middle: Percentage of depth penetrated by amoebae to total tissue thickness. Inset: Distributions of parasite *z* position over tissue height at different times WP (0 μm, basal side). *n* = 4. Bottom: Tissue connectivity. *n* = 6. (**C**) Fixed images of the invasion. Left: 1 hour. Right: 3 hours. Top: Amoeba lysing brush-border actin and E-cadherin. Bottom: Phagocytosis of a dead Caco2 cell (left) and dead cells within the tissue (right). Scale bars, 20 μm. (**D**) Tissue connectivity (left) and dead cells (right) (7 hours, live): Control tissue, with amoebae, WP and WOP, and with a cysteine protease inhibitor (E64). Unequal-variances *t* test. *: 0.05; **: 0.01; ***: 0.001; ****: 0.0001. (**E**) Cysteine protease activity is necessary for invasion. 3D reconstruction of slices imaged as confocal *z* stacks after 7 hours of infection. Amoebae with (right) and without (left) E64, WP (top), and WOP (bottom).

**Fig. 6.**
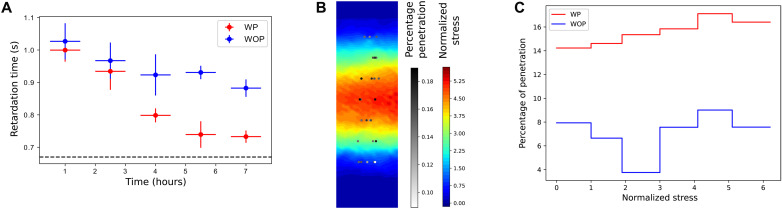
Mechanobiology of tissue invasion by *E. histolytica*. (**A**) Live WP-WOP comparison of the retardation time of the tissue infected with *E. histolytica* over time. Dashed line corresponds to the chip without tissue. *n* = 6. (**B**) Stress map (averaged over a cycle) with the percentage of penetration depth at 6 hours superimposed; the *x* axis of marked points is meaningless because penetration is averaged over the *x* axis for additional accuracy. (**C**) Histogramic relation showing the correlation between stress and percentage of penetration WP compared to a WOP proxy where the WOP distribution is used on the WP stress map.

The combination of novel methodology to study deformable OOCs live can be summarized as follows (Fig. 1): Image the chip infected with either of the two pathogens, reconstruct the moving tissue in 3D (+ time) from desynchronized 2D sequences, extract the surface of the tissue as a manifold, project the manifold onto 2D for visualization while respecting the real deformation in 3D, eliminate the movement in the *z* axis because it does not induce any mechanical deformation and complicates visualization (Fig. 2), measure the remaining movement within the tissue to compute a map of the stress therein (Fig. 3), and compare the spatial tissue tension with local measures of infection (Figs. 4 to 6). The measures of infection are tailored to each of the pathogens and are detailed in Materials and Methods, for example, bacterial colonization, activation of virulence factors, and parasite penetration.

#### Tissue reconstruction and visualization

At 0.15 Hz, the frequency of physiological peristalsis is too high in comparison to the acquisition speed of spinning-disk confocal microscopes (~8 to 32 s per stack). In combination with the thickness of the tissue (~200 to 300 μm), it prevents live 3D imaging in deformable OOCs. The same motion complicates 2D imaging too because it introduces an orthogonal component that constantly drives the tissue out of the focal plane (Fig. 2A and movies S1 and S2). A third problem is the curvature of the tissue, which introduces axial (*z*) variations in the focus and practically halves the field of view (Fig. 2A, “curves”).

To study host-pathogen dynamics, we first aimed at reconstructing the tissue in 4D. To this end, instead of acquiring standard *z* stacks, we programmed the microscope to acquire a continuous 14-s (~2*T*) movie at each *z* position (Fig. 2A). With the resulting video data, we tackled the challenges above by developing three ad hoc methods: alignment, manifold projection, and motion correction (Fig. 2B and movies S3 and S4).

##### Alignment

The acquisition of each *z*-slice movie starts at a different phase of the peristaltic cycle and is therefore not synchronized with the rest. It was thus necessary to align the videos in time to reconstruct the tissue. Similar approaches have been used before to study the fast-beating heart of the zebrafish (*38*, *39*), but the problem specifications are different enough to deter the application of these solutions. To align the videos in a way that is faster and better suited to our data, we formulated a Kalman filter (*40*) that combines two different measures of synchronization. The first measure is relative and therefore accumulates errors: To recover the time lag between two consecutive *z*-slice videos, we find the set of frames that best match one another by maximizing their statistical interdependence. Albeit less precise, the second measure is absolute and can therefore compensate for the error accumulation: In this case, the lag is measured by directly identifying the cyclic phase of each video according to the periodic motion of the intensity center. The Kalman filter puts together both measures in a way that minimizes the final error, resulting in smooth 3D videos of the moving tissue or any other fluorescence channel. See Materials and Methods for the mathematical formulation of this novel approach.

##### Manifold projection

Once the tissue is reconstructed in 3D, the next aim is to straighten out any curvature so that it can be visualized in a single 2D frame. To this end, we use the wavelet transform (*41*) to capture the medial axis of the epithelia in the 3D intensity profile and subsequently fit a manifold (*42*) to reparameterize the shape of the tissue in only two dimensions. The resulting curvature profile shows that the tissue slants lengthwise at a rate of 3 μm every 100 μm, explaining the blur observed in confocal slices (Fig. 2). Using this manifold, we can compute the proper distances to project the 3D tissue in a way that brings each portion of the surface into focus while accounting for the non-Euclidean metric. Applying the same transformation to the other channels allows us to observe how the pathogens move on the tissue too. See **M**aterials and Methods for the mathematical formulation.

##### Movement correction

Even if the tissue projection then lies completely on focus in a single frame, the motion induced in the *z* direction by the peristaltic cycle brings it constantly out of plane. By fitting a sinusoid function on the orthogonal movement, we can subtract it from the 2D manifold projection. In this way, the 2D movie stays on focus all along the peristaltic cycle, and only the in-manifold movement remains, allowing us to isolate the deformation of the tissue and, in the coming sections, the parasites’ migration too. See Materials and Methods for the mathematical formulation.

#### Tissue rheology and mechanics

While the image analysis methodology presented above allows observing and quantifying the dynamics of deformable OOCs live, the encasing of the chips complicates studying any physical cues therein. In the context of host-pathogen interactions, for example, we wanted to measure the stress of the tissue at a small enough resolution to allow comparisons with the success rate of single parasites during infection. To study the mechanics of the tissue inside the chip, we leveraged our 4D live imaging framework to develop three further analytical methods (Fig. 3A): We first studied the rheology of the tissue to propose a physical model of its behavior; we then developed a method to extract the movement of the tissue and compute local stress according to this model; lastly, we built a custom hypothesis test to assess the correlation between local stress and local virulence.

##### Viscoelastic constants

Epithelial tissue has been repeatedly reported to behave viscoelastically (*43*). In particular, the standard linear solid (SLS) model combines viscous dampers and elastic springs in 0D to model the polymer-like response of tissue layers such as those present on chips (Fig. 3B, schematic) (*44*). In our case, however, the presence of the chip encasing prevents the use of any rheological probe, while the PDMS scaffold alters the mechanical behavior of the tissue with respect to the pressure pump. In the rheological analysis presented in Materials and Methods, we characterize the SLS model by presenting OOC-friendly ways to measure two of the constants therein, namely, the retardation time constant and Poisson’s ratio, whereas Young’s modulus and the relaxation time constant remained inaccessible. Notice that in the SLS, the time constants characterize the time scales of viscous energy dissipation, which describe the polymeric disentangling that delays the purely elastic responses. In practical terms, this means that upon releasing the pressure, the tissue does not go back to its resting state instantly but rather unstretches progressively (Fig. 3B).

Although the stationary response of the tissue to pressure increments was linear as expected from the SLS, the optical resolution was not enough to decouple the effect of the much more rigid PDMS on the Young’s modulus from that of the softer tissue (fig. S1, B and C). Similarly, the relaxation constant was inaccessible because we were unable to fix the width of the chip. However, we did manage to use the pressure pump to both measure the retardation constant and decouple it from the PDMS response (fig. S1A). In particular, we found that the retardation stayed constant in healthy tissue (1.02 ± 0.03 s at 0 hour, 1.00 ± 0.04 s at 2 hours, means ± SD), whereas we recovered the range of values typical to PDMS (*45*) upon trypsinization of the chip (0.68 ± 0.02 s at 2 + 1 hours, *P* = 1 × 10^−6^). See a complete physical analysis in Materials and Methods. The retardation constant characterizes the creeping behavior of the epithelia and, in our case, constitutes the most interesting property because it reflects the degree of cross-linking within a polymer (*45*). Aside from characterizing the stress, this measurement doubles as a probe for the state of the tissue.

##### Velocimetry and continuum model

To measure the displacement within the epithelial layer, we adapted a technique called optical flow (OF) (*46*), which extracts motion from video sequences by following the brightness of pixels, to work on the 2D manifold sequences by adapting the inner products and derivatives to the tangent bundle; in this way, the proper angles and distances of the curving tissue are taken into account, much like a cartographer does to draw a 2D map of planet Earth. However, the resulting displacement does not only measure deformation but also translational and rotational movement, which do not induce any stress in the tissue. Therefore, to only capture its relative deformation, we computed the strain of the tissue as the symmetric gradient of the displacement. We then extended the viscoelastic model characterized above in 0D to 2D tensorial continuum mechanics to establish a relation between the strain and the stress across the whole tissue (see Materials and Methods). Together, the modified OF and the continuum model complement each other on-manifold to yield spatiotemporal maps of deformation and stress (Fig. 3, C and D, and movies S5 to S8). For better accuracy, the two methods are coupled together in a unified framework. The spatial distribution of these maps is independent of the elastic constants, whereas the time constants only introduce a phase lag between strain and stress (see the “Complex analysis” section), which is also irrelevant to our subsequent correlation studies. See mathematical formulation in Materials and Methods.

##### Hypothesis testing

To assess the statistical significance of the correlation between the spatial distribution of mechanical tension and the measures of invasion success, we developed a custom hypothesis test. This test is an adaptation of the classic image-point or image-image correlation tests (*47*, *48*). However, instead of assuming homogeneity as a null hypothesis, we used a nonhomogeneous distribution that is based on the results of the nonstressed control condition. In this way, the test directly informs us of whether the correlation is significant with respect to our particular control. In addition, the point correlations are weighted by each of the measures, for example, parasite penetration or activation time of virulence markers. See mathematical formulation in Materials and Methods.

Together, this methodology constitutes a powerful asset to study dynamic and mechanical processes in deformable OOCs. Here, we use it to study the invasion of intestine-chips under peristalsis by enteric pathogens.

### Revealing the dynamics of pathogen invasion using image processing and mechanobiology

The development of the live imaging and mechanobiology methods above allowed us to study and quantify the early stages of infection of two models: *S. flexneri* and *E. histolytica*. To this end, each of the pathogens (with its corresponding fluorescent label) was introduced at the apical side of the intestine-chip (matured with Caco2–E-cadherin–GFP) and imaged under a spinning-disk confocal microscope (see Materials and Methods). During the experiment, peristalsis was maintained by applying a lengthwise periodic stretch (10% at 0.15 Hz). To single out the effect of the induced peristalsis on the infection, we also studied motionless chips (e.g., Figs. 4 and 5). We will refer to these latter chips as without the peristalsis-like stretch (WOP) and to the former as WP. For further comparison, we set up a pair of infection-impairing conditions: an avirulent strain of *S. flexneri* and the addition of an inhibitor for *E. histolytica*; these have allowed us to study the differential effect of peristalsis when the pathogens are unable to exploit some of their invasion mechanisms.

Once the imaging session finished, the reconstruction and visualization algorithms (see the “Image processing and mechanobiology for deformable OOCs” section) were used on each of the multiple channels to yield 3D sequences and their corresponding 2D projections. Image analysis was then used to quantify the infection at the tissue scale according to several pathogen-specific measures (see Materials and Methods). The stress maps were computed from the reconstructions too (see the “Tissue rheology and mechanics” section) and compared to local versions of the virulence measures with the aim of uncovering potential correlations (see Materials and Methods). They were also compared to the infection-impairing conditions. Each experiment was repeated WP and WOP multiple times.

#### Dynamical invasion of S. flexneri

To study the invasion by *S. flexneri*, we introduced a wild-type strain of the bacteria expressing mCherry (*Shigella*-WT-mCherry) into the intestine-chips. We imaged the evolution of the infection during 1 to 2 hours at different zones of the chip (see Materials and Methods). The resulting sequences were then processed using the methods described above. The invasion was compared to that of an avirulent strain (*Shigella-mxiD*), WP and WOP, as well as to noninfected chips. To quantitatively assess how bacteria invade the tissue from the reconstructed videos, we defined two measures: the extent of bacterial colonies and the individual time of penetration into host cells (Fig. 4A; see Materials and Methods). To probe the latter, we used another strain named *Shigella*–red fluorescent protein (RFP)–TSAR that expresses GFP upon T3SS activation through a transcription-based secretion activity reporter (TSAR) (*49*). In the case of TSAR experiments, a blue membrane probe [Pro12A (*50*)] together with standard Caco2 cells was used in substitution of the Caco2–E-cadherin–GFP cell line (fig. S2F).

##### Peristalsis boosts bacterial colonization and T3SS-mediated cell-to-cell spreading

We first observed that bacterial colonization not only started earlier under peristalsis when compared to the nonstretched control but also expanded much more rapidly thereafter (Fig. 4B). In particular, bacteria expanded at twice the speed WP as they did WOP (slope ratio of 2.05 ± 0.79 at 2 hours; Fig. 4D). Within the hour, however, the tissue pockets harboring *Shigella* start reaching capacity, and bacterial colonization slowly “converges” to increasingly similar sizes under both conditions. This behavior is in agreement with our previous data obtained 1 hour after infection on fixed samples (*17*). This first step appeared as a passive process independent of the virulence of *Shigella* as bacteria devoid of the capacity to secrete effectors into the host cells (*Shigella-mxiD*, avirulent strain) showed similar colonization patterns to those of wild-type bacteria both WP and WOP (growth slope ratio was not significantly different; Fig. 4D).

Accordingly, while actin was recruited by the epithelial cells during infection with wild-type bacteria, tissue challenged with the *Shigella*-*mxiD* strain did not recruit new actin (fig. S2E) and therefore showed weaker colocalization with growing bacterial colonies (Fig. 4D). In the TSAR strain, actin spots were also found to colocalize with actin foci (Fig. 4C) but showed especial affinity for bacteria that had recently activated their secretion system (Fig. 4, C and D).

During the first 2 hours of infection, TSAR activation passes through two distinct phases to eventually reach a final steady-state phase as bacteria proliferate and spread within the tissue (Fig. 4C). The first phase shows no activation because bacteria are only starting to sediment on the tissue; this delay is common to both conditions and might reflect either the inherent activation time of the GFP compound in the TSAR strain or the time bacteria take to actually start up their infection mechanisms. A second phase in the infection shows how the T3SS is engaged (TSAR activated seen by red and green bacteria) at a time where the bacteria are adhering to the cell and colonies are still growing (Fig. 4C, label 1). Then, a transient stage shows internalized bacteria shutting off T3SS (red bacteria, Fig. 4C, label 2). In addition, the third and last phase reflects activations dominated by cell-to-cell spread (Fig. 4C, label 3). While bacteria WP see their activation rate enhanced in the second and final phases as compared to those WOP (even when correcting for the initial increase in colonization speed; Fig. 4B), the third phase is the most interesting as it best reflects the speed of cell-to-cell spread. The fact that the activation of GFP is only temporary and that the activation ratios (Fig. 4B) plateau in due time (at 4% WP and 2% WOP) signifies that the GFP signal is being constantly renewed. Therefore, the ratios reflect the speed of cell-to-cell spread, which finds a steady state at the plateau; this speed is doubled WP (Fig. 4B, inset), which explains why the invasion appears much more advanced upon fixation at 3 hours and why the tissue is overall more damaged (fig. S2D). Notice that these ratios might be underestimated because not all bacteria counted in a colony are in contact with the tissue and thus able to penetrate the epithelial cells. For example, bacteria might be counted cubically (quadratically in 2D), while surface available to injection is quadratic (linear). Conversely, tissue infected by the *mxiD* strain did not present significant damage within the same time frame because bacteria could neither penetrate the tissue nor spread through host cells. In conclusion, the peristaltic cycle not only increased the virulence of the colonization and penetration process but also accelerated the onset of infection by promoting bacterial sedimentation.

##### Local mechanical stress accelerates T3SS-induced cell-to-cell spreading

We then asked whether it was the motion of the tissue that fostered the infection by multiplying the probability of successful invasions. On the one hand, the cyclic movement did not seem to promote cross-colony migrations in *S. flexneri* because no bacterial transfer was observed after the initial sedimentation. On the other hand, bacteria jumped from cell to cell at double the WOP speed. While the differential colonization speed between conditions appeared as a passive effect of the movement, we wondered whether (being already within the depths of the tissue) the faster cell-to-cell spread under WP conditions could be a result of higher stress in the tissue junctions. Therefore, we studied the effects of local mechanical stress on *S. flexneri* by comparing the speed of TSAR activation of individual bacteria. To this end, we computed stress maps of the tissue using the blue membrane probe Pro12A (*50*) to avoid fluorescent channel leaks between E-cadherin and TSAR activation (fig. S2F). As shown in Fig. 4 (E and F), we found that bacteria were activated significantly sooner where epithelial stress was higher (*P* = 0.00012; see Materials and Methods).

#### Dynamical invasion of E. histolytica

We studied *E. histolytica* infections by inoculating (10^6^ amoeba/ml) intestine-chips preincubated with a probe for dead cells (DRAQ7; see Materials and Methods) to observe the consequences of amoebic infections in real time. The chips were then imaged during 7 hours at intervals of 30 min (early stage) to an hour (late) at three different zones (see Materials and Methods), and the infection sequences were reconstructed. The invasion was compared to that in the presence of a cysteine protease inhibitor (E64), WP and WOP, as well as to noninfected chips. The infection measures for *E. histolytica* were chosen to quantify cell death, tissue connectivity, and trophozoite penetration [Fig. 5, A (i to iii) and B; see Materials and Methods], all markers of the virulence of amebiasis. These measures were repeatedly taken over time as the infection advanced.

##### Peristalsis boosts amoeboid invasion and tissue degradation

In the presence of the parasite, we observed that the tissue WP goes from healthy to a death rate of around 10% over the course of the first 3 hours and stalls slowly thereafter to reach 17% at 7 hours (Fig. 5B). During this time, the tissue junctions disconnect progressively with an inflection point at around 3 to 4 hours after challenge, when the connectivity decays markedly in a sort of collapse or failure (Fig. 5B). This view of the infection timeline allowed us to more precisely follow the evolution of the tissue by means of a fixing protocol. We observed that *E. histolytica* degraded the actin on the brush border, phagocyted dead cells, and cleaved E-cadherin junctions during the process of infection (Fig. 5C). We also confirmed the 3- to 4-hour range as a key point as regards the deterioration of both the E-cadherin junctions and the actin brush border covering the apical epithelial layer, as well as a sudden increase in cell death (fig. S2A).

Without mechanical stretch, tissue destruction decreased substantially (Fig. 5B and fig. S2A), reaching a dead-cell ratio of only 8% at the 7-hour mark as compared to the 3% score of the parasite-less control (Fig. 5D). However, the onset of the infection was quicker in the absence of peristalsis. Similarly, the connectivity of the tissue was less affected WOP (Fig. 5, B and D). To test the role of the lytic activity in the invasion, we added a cysteine protease inhibitor (E64) upon infection. Under the blocking effects of E64, death and connectivity measures returned to levels only slightly higher than those of the noninfected controls, while the difference between the WP and WOP conditions became insignificant (Fig. 5D). Accordingly, the E-cadherin junctions and the brush border remained practically intact (Fig. 5E). This confirms that the cysteine protease activity is necessary to effectively degrade and invade the tissue on the chip. We further investigated the degradation of the cross-linkers within the tissue by measuring the viscoelastic retardation constant during infection. We observed that the time scale shortened progressively under both conditions, but the tissue WOP lost its viscous properties significantly slower (Fig. 6A). In short, the peristaltic cycle boosted the virulence of *E. histolytica*.

We then tracked the penetration of amoebae (Fig. 5A, i and ii), which increased steadily during the experiment. In 6 hours, the average parasite penetrated around 15% of the tissue’s thickness with the help of the peristalsis-like cycle, whereas amoebae WOP only made half their way in (around 7%; Fig. 5B). Penetration appeared easier WP independently of the lysate action (notice that connectivity is more delayed than penetration with respect to WOP).

We also followed the parasites in *x*-*y* (fig. S2B). WOP, their movement on the plane was characterized by a random phase with different diffusivities at the beginning of the infection (fig. S2B) that halted after an hour, after which the only remaining movement was directed toward the bottom of the tissue (in *z*). More precisely, during the first hour, part of the parasites appeared superdiffusive, traversing and exploring the tissue until settling down at a specific spot, but most were subdiffusive, likely as a result of physical constraints within the tissue.

Although they start their downward motion sooner (Fig. 5B), amoebae under WP conditions were found to be much less motile on the plane (fig. S2B), with a higher population belonging to the subdiffusive regime. The distribution of the *E. histolytica* penetration was very wide WP (Fig. 5B, inset), indicating different success rates of invasion even within the same chip.

##### Local mechanical stress enhances amoeboid penetration

Tracking the displacement of *E. histolytica* trophozoites showed that their migration was predominantly directed in the *z* direction. In addition, amoebae appeared more successful (albeit heterogeneously so) at penetrating the tissue WP despite similar starting distributions (fig. S2B). We therefore speculated that the heterogeneity of the *E. histolytica* population could reflect the spatially heterogeneous stress within the tissue. To study whether local stress facilitates *E. histolytica* invasion, we computed the stress maps and tested them against the speed of penetration of individual amoeba at different positions in the tissue. We found that the parasites were statistically more successful in zones of higher stress as amoeba penetration showed significant correlation (*P* = 0.0038; see Materials and Methods) with local membrane tension (Fig. 6).

#### Comparison of the invasion of S. flexneri and E. histolytica

Although the virulence of both pathogens is bolstered by the peristaltic motion, together, the measurements above highlight two completely different invasion strategies. Tissue destruction is much faster overall with *E. histolytica* (7 hours), due to its known lysing activity; *Shigella* only reached comparable levels of tissue destruction overnight (15 hours). Conversely, *Shigella* effectively multiply over the course of infections, whereas *E. histolytica* do not. In addition, movement has an adverse effect on the onset of amoeboid infection but appears advantageous for enteric bacteria. While the movement of the tissue is responsible for the differential onsets of the infection, the increased success of the pathogens at spreading within the tissue WP is independent of their initial condition. In the absence of flow and other factors, this reveals that the stress endured by the tissue plays a role in facilitating the invasion of the pathogens and the resulting tissue destruction. Accordingly, the measures of pathogenicity are statistically enriched in zones where host cells are under tension: *E. histolytica* penetrates more easily and *S. flexneri* spreads from cell to cell more rapidly in hotspots of tissue stress. These correlations introduce mechanical forces as a key factor of pathogen invasion while distinguishing them from mere passive effects related to other factors such as movement.

## DISCUSSION

Because of technical constraints, deformable OOCs are seldom studied live. However, our unprecedented look at early-stage infection under physiologically relevant conditions confirms the importance of quantitative bioimaging as a vehicle of discovery in complex and high-dimensional datasets (*51*). To foster this direction, we are now working to make our computational developments available to the OOC community. In a similar way, this study also aims at establishing a platform for rheological and mechanobiological studies. The study of physical cues in biological function has recently been key to many discoveries (*52*): from the organ scale [for example, in development, where our approach could shed light on the mechanical interplay involved in the formation of crypts (*53*)] to the nanoscale [for instance, micropipette experiments have shown that severe acute respiratory syndrome coronavirus 2 spike recognition is strengthened under tensile forces of the order of 10 pN (*54*)]. By leveraging the proposed framework together with objectives of different magnifying power, this type of studies could now be repeated under more physiologically relevant conditions. In the present work, this methodology has allowed us to explore the importance of peristalsis in host-pathogen interactions within the gut.

For *S. flexneri*, the peristaltic cycle boosts the colonization of the crypt-like structures of the colon. This is particularly noticeable during the first hour of infection and could help bacteria—which are devoid of any adhesins or migration mechanisms—escape immune surveillance. Our results indicate that the virulence of *Shigella* is also enhanced by peristalsis, at least partly due to local tissue stress. Membrane tension has been reported to reorganize plasma membrane components like lipids and F-actin (*55*). As regards *Shigella*’s invasion and cell-to-cell dissemination, F-actin and cholesterol are known to be key host factors that are massively recruited to bacterial foci. The cyclic stretching of the tissue might facilitate a local disorganization of F-actin that can then either be more easily hijacked by *Shigella* or enable closer contact between the bacteria and the plasma membrane, thereby triggering the expression of virulent genes and potentially explaining the twofold increase in spreading rate.

As opposed to sedimenting into the crypts like *S. flexneri*, *E. histolytica* starts by binding to the apex of the epithelium because they do have robust adhesion mechanisms to human cells. To this end, amoebae express multiple molecules involved in parasite-cell interactions such as lipopeptidophosphoglycan; lysine and glutamic acid–rich protein 1; and serine-, threonine-, and isoleucine-rich protein (*56*–*58*). Peristalsis increased the speed and depth of parasite penetration to the detriment of apical migration. WOP amoebae were thus able to lysate a bigger portion of the cells at the onset of invasion due to higher apical migration, thereby ruling out that cyclic motion simply increases the spread of the infection. It is possible that *E. histolytica* could sense mechanical cues that regulate its migration on the epithelium and its cell-killing activity. The correlation between local tension and penetration suggests that the parasites could preferentially bind membranes under tension while profiting from better access to the perhaps-fragilized cell junctions for lysing. Similar mechanosignaling has been described before in vitro (*5*, *59*).

Much like in ex vivo human explants (*1*), cysteine activity appears essential for tissue invasion in intestine-chips. Under peristalsis, the parasites quickly bring the tissue into collapse. The cyclic stress seems to help them reach deeper while weakening the junctions under increased tension, thereby increasing the effects of the lysing activity in two complementary ways and suggesting an additional mechanical influence of the peristalsis on the penetration phase. In this context, it remains to be explored how the dynamic responses of the parasites and the host cells—e.g., through transcriptional regulation or secreted factors—combine with mechanical stress to increase the virulence of the destruction caused by *E. histolytica* under peristalsis.

Peristalsis is known to participate in the renewal of gut tissue by shedding epithelial cells and microbiota (*60*). Inhibition of gastrointestinal motility typically favors bacterial overgrowth, showing that, in principle, peristaltic motion is an important factor to limit the risk of infection. However, our study shows that this physical cue is determinant for the invasion of both *Shigella* and *E. histolytica*, which differ greatly in size, life cycle, and mechanism of infection but share a common predilection for the human colon as opposed to the rest of the gut. It appears that the two pathogens have evolved to exploit the colonic environmental cues, even if it may seem counterproductive at first glance. Therefore, our work invites to reconsider the way we should investigate host-pathogen interactions in the niche of their target organ.

## MATERIALS AND METHODS

### Cell culture and pathogen strains

Caco2 cells (clone TC-7) were obtained from the American Type Culture Collection. Cells were grown in Dulbecco’s modified Eagle’s medium (DMEM; Gibco) supplemented with 20% fetal bovine serum (Biowest) and nonessential amino acids (Gibco) in 10% CO_2_ at 37°C. To generate the gene-edited strain Caco2–E-cadherin–GFP, we used the CRISPR-Cas9 technology to insert enhanced GFP at the 3′ end (position 68833480) of the *CDH1* gene through the following guide RNA sequence: AAGCTGGCTGACATGTACGG. Production of the protein E-cadherin–GFP in the edited strain was verified by Western blot and polymerase chain reaction. The strain and cells are available upon request.

We used the wild-type derivative *S. flexneri* 5a (M90T) (*61*) and its *mxiD* mutant derivative (*62*). Both these strains harbor the virulent plasmid encoding for mCherry. We also used a TSAR strain, which was previously described in (*49*). Bacteria were grown at 37°C on trypticase soy broth (TSB; Becton Dickinson) agar plates containing 0.01 Congo red (Serva). Individual colonies were grown at 30°C overnight with ampicillin. From this culture, the pathogens were grown at 37°C in TSB to obtain a suspension of 10^10^ colony-forming units/ml. Before infection, bacteria were washed three times in Dulbecco’s phosphate-buffered saline (PBS) and diluted to the desired multiplicity of infection in DMEM supplemented with Hepes (20 μM; Gibco).

We used the pathogenic *E. histolytica* HM1:IMSS strain. The trophozoites were cultured axenically in TYI-S-33 medium at 37°C (*63*). Preceding the day of infection, *E. histolytica* were incubated overnight at 37°C with CellTracker Red CMTPX (2.5 μM; Thermo Fisher Scientific). The fluorescently labeled amoebae were then washed with warm incomplete (serum-free) TYI-S-33 (iTYI) and resuspended in iTYI (37°C).

### Human intestine-chip

Intestine-chips were cultured from empty S1 chips (Emulate) made of PDMS in the Human Emulation System provided by Emulate (Boston, MA, USA). The intestine-chip production protocol consisted of an activation phase followed by ECM coating as per the manufacturer’s instructions. In short, chips were activated using a mix of ER-1 and ER-2 solution (Emulate) and exposed under ultraviolet light (36 W, 365 nm) for 20 min. Then, chips were rinsed with ER-2 solution followed by PBS (Gibco). Next, the coating was applied by incubating an ECM solution [Matrigel (100 μg/ml; Corning) and rat tail collagen type 1 (30 μg/ml; Gibco) diluted in PBS (Gibco)] overnight at 4°C and 1 hour at 37°C and 5% CO_2_ before cell seeding. Cells were seeded at a concentration of 1.5 million/ml in the upper channel for 2 hours at 37°C and 5% CO_2_. After cell attachment, the upper channel was gently washed using warm cell culture medium supplemented with penicillin (100 U/ml) and streptomycin (100 μg/ml). Cells were then cultured overnight on the S1 chips without perfusion. Last, the chips were connected to the Pod-1 (Emulate) and placed in the Zoë CM1 (Emulate); they were kept there for the duration of the culture. The following culture conditions were applied: 37°C, 5% CO_2_, a flow rate of 30 μl/hour, and 10% of lateral mechanical stretching at a frequency of 0.15 Hz. The culture medium was refreshed every 48 hours.

### Intestine-chip infections

Depending on the pathogen, the chips were incubated immediately before infection with the applicable subsets of the following probes:

1) With *E. histolytica*. Before infection, both channels of the chip were filled with warm iTY (37°C) containing DRAQ7 (a far-red dye that only stains the double-stranded DNA of dead or permeabilized cells; 1/50, 6 mM final; Abcam) and, occasionally, E64 (cysteine protease inhibitor; 100 mM; Alfa Aesar). As a preliminary measure, the chip was then immediately inspected under the microscope for 10 min at 37°C. Once ready for infection, the chip was temporarily removed from the microscope, and its top channel was filled with a warm solution of iTYI containing 10^6^ trophozoites/ml together with DRAQ7 (and occasionally E64). The chip was then put back under the microscope (37°C) and connected to the microfluidic pump.

2) With *S. flexneri*. Before infection, both channels of the chip were filled with warm medium having one or more of Pro12A (blue membrane dye; 2 nM), SiR-Actin kit (0.2 nmol; Spirochrome), and lectin (peanut agglutinin) AF647 (1/500; stock solution: 3 mol of dye/mole; far-red dye).

Pro12A is a noncommercial membrane marker that allows labeling the membranes of the epithelial cells in blue with better specificity and less channel leaks when compared to commercial dyes. It was provided by the laboratory of A. S. Klymchenko (*50*).

### Live microscopy and pressure control

During the lapse of infection, the intestine-chips were imaged using a T2i spinning-disk confocal microscope by Nikon with a Hamamatsu ORCA-Flash 4.0 digital complementary metal-oxide semiconductor (CMOS) camera, although a Photometrics Prime 95B sCMOS camera was used occasionally. Depending on the aim, objectives were either a 10× [Plan Apochromat 10× DIC N1 with a numerical aperture (NA) of 0.45] or a 20× (Plan Apochromat Lambda 20× with an NA of 0.75); objectives of higher-magnification power did not have enough working distance to image through the bottom channel of the chip. The microscope was programmed using the JOBS environment of the proprietary NIS Elements software by Nikon (see NIS-Elements_JOBS_and_HCA). We chose confocal microscopy for its accessibility: Lattice light sheet microscopes have better penetration and speed (although still not fast enough for 3D) but are not compatible with the chip dimensions unless specifically modified to this purpose.

Pressure in the vacuum channels of the chip was controlled with a Fluigent microfluidic pressure controller (Flow EZ, LineUp series). To mimic peristalsis, a sinusoid pressure was fed to the chip vacuum chambers with a period of 6.6 s and an amplitude of −350 mbar (oscillating from 0 to −700 mbar), amounting to a maximal tissue stretch of around 10%. Other pressure settings were used when performing the rheological experiments. The pump was programmed using the Microfluid Automation Tool software by Fluigent. At times, an Elveflow microfluidic controller (OB1 MK3+) coupled to a vacuum pump was used to the same effect. During live imaging, the medium perfusion was stopped in both upper and lower channels of the intestine-chip.

### Fixed assays and immunofluorescence

After live imaging, the chips were fixed using a paraformaldehyde solution 4% (Thermo Fisher Scientific). Thirty minutes later, all channels were flushed with PBS. Using a vibratome (Thermo Fisher Scientific, Microm HM 650 V), the chip was then cut into crosswise slices of roughly 200 mm, which were, in turn, permeabilized for an hour at room temperature (RT) using a buffer [PBS/0.2% Triton X-100/0.5% bovine serum albumin (BSA)].

The transversal sections in fig. S1 were first permeabilized with a 1% solution of saponin (Sigma-Aldrich) in 1× PBS for 1 hour at RT. They were then washed three times with PBS and incubated with a 2% solution of BSA (Sigma-Aldrich) in 1× PBS (2 hours, RT). After washing, the slices were incubated with SiR-Actin diluted 1/1000 in a 1× PBS solution of 2% BSA and 1% saponin (1 hour, RT). The resulting samples were washed an additional three times and conserved in PBS at 4°C. We remark that SiR-Actin binds F-actin, which is present inside the brush border of the microvilli. All prepared sections were imaged using the same confocal microscope described above, alternating between a 20× and a 40× (CFI Apochromat Lambda S 40XC water immersion with an NA of 1.25) objective.

### Quantitative measures of infection based on image analysis

At each time point, all quantitative measurements described hereafter were consistently taken at three different zones of the chip, unless stated otherwise. Those were the center of the chip and the two regions adjacent thereto (both sides) without overlap. Conversely, the number of biological replicates is defined as “*n*” in each experiment, for the number of chips, when quantitative data are referenced.

To study the death of host cells induced by the pathogens, cells were dyed in live experiments with DRAQ7 (far-red emission). After due imaging, we segmented the dead cells on the far-red channel at each time point of the reconstructed invasion video. Segmentation was performed using a *k*-means clustering algorithm. The number of pixels corresponding to dead cells at each time point was then divided by the total extension of the tissue to yield the final percentage. To make sure that it was not the pathogens that died, we checked for colocalization on the red channel, which corresponded to the pathogen’s marker. In this way, we also saw that the number of *E. histolytica* remained constant, i.e., they did not seem to multiply.

To study tissue connectivity, we began by imaging the intestine-chip cultured with the Caco2–E-cadherin–GFP strain. We continued by processing the resulting signal with an edge-preserving nonlinear anisotropic filter. The choice was Catté’s extension of Perona-Malik’s diffusion, albeit we obtained equivalent results with a denoiser based on anisotropic total variation (*64*, *65*). The aim of the filter was to denoise the image in a way that preserves the piecewise constant nature of the E-cadherin junctions around the membrane, which vaguely resemble a honeycomb-like network (*65*, *66*). To detect the cadherin edges in the postfiltered image, we first applied Sobel’s operator. We then thresholded the resulting edge intensity image using a clustering algorithm and counted the number of disjoint groups, which corresponded to discontinuous pieces of membrane. The total number of cadherin pixels (above the threshold) was then divided by the number of disjoint groups, yielding an “average” measure of pixels per group. In damaged tissue, E-cadherin junctions disconnect as cells break apart, creating more disjoint groups and thus lowering the connectivity measure. To further relativize connectivity, we expressed the value as a percentage by dividing it by the very same measure applied to the tissue before challenge. As a remark, in our case, while the combination of the anisotropic filter and the inherent smoothing of the Sobel filter was enough to bring out the edges for detection, other images (especially those taken with higher-magnification objectives) may benefit from more robust edge detectors such as Canny’s, albeit preferably enforcing anisotropic smoothing.

To track *E. histolytica* trophozoites, we first detected the location of each parasite at each time point using a spot detection (SPD) method based on the stationary wavelet transform as described in (*67*). We then used an MH algorithm to join the detections into trajectories over the entire sequences (*67*, *68*).

To monitor the penetration of the parasites, we projected the reconstructed images on the tissue’s cross section in the form of histograms and followed the parasites thereon. To yield a more robust statistical analysis, in Fig. 5, the penetration was averaged lengthwise and divided into crosswise regions.

To assess the degree of colocalization between actin recruitment and TSAR activation during bacterial infection, we first detected the actin and TSAR spots in their respective channels. We then performed a statistical distance analysis between the resulting spots using Ripley’s *K* function following (*69*).

We measured bacterial colonization by counting the number of pathogens using the SPD method at each time point of the video sequences. Alternatively, the total area of bacterial colonies as detected by *k*-means clustering followed the same growth pattern as the population detected with SPD.

We detected TSAR activation on the corresponding green channel using a hierarchical *K*-means clustering algorithm when quantifying activation in bulk for Fig. 4B. For the analysis in Fig. 4E, we required better localization of the TSAR activations, and thus, we detected and tracked them with a combination of SPD and MH.

The stress-measure correlations performed in the “Revealing the dynamics of pathogen invasion using image processing and mechanobiology” section and Figs. 4 (E and F) and 6 were performed over different positions of four different chips for each of the pathogens. In the case of *E. histolytica*, the penetration was averaged lengthwise and divided into crosswise sections. This had the aim of increasing statistical robustness because the *z* axis has poorer resolution. The TSAR activations were counted as spots (SPD) and then tracked (MH) to make sure that each activation was only counted once until it vanished; around 2800 activations were considered.

Unequal-variances *t* tests were used for Figs. 4D and 5D. Some of the algorithms were used as plugins in the Icy platform (*70*) (SPD, MH, anisotropic filter, and Ripley’s *K*; icy.bioimageanalysis.org); the rest were implemented in Python.

### Methods on image processing and mechanobiology

#### Alignment

The scanning unit of the microscope is too slow to keep up with the peristaltic motion of *T* = 6.6 s in 3D: Although the tissue is around 80 μm thick, capturing the tissue in motion together with the infectious agent requires a *z* range of roughly *r* = 150 μm, which is equivalent to *N* = [*r*/∆*z*] = 37 slices at a resolution of only ∆*z* = 4 μm; we index by *Z* = {0. . *N*} the resulting *z* positions collected in {*z*∆*z* ∣ *z* ∈ *Z*}. In contrast, nonphototoxic exposure times range in ∆*t* ≈ 0.3 s, making each *z* stack much longer than a single period and thus leaving the top and bottom images at completely different phases of the motion.

To image the 3D tissue live, we try to exploit the periodicity of the motion. We programmed the microscope to automatically acquire a temporal sequence *I_z_* : Ο × *T_z_* → ℝ_≥0_ spanning a time range (*T_z_*) = 2*T* at each of the consecutive *z ϵ Z* positions, where Ο is the microscope’s field of view, and we expect the signal to be periodic *I_z_*([*t* + *T*]*_T_z__*) − *I_z_*(*t*) ≈ 0 ∀ *t* ∈ *T*_z_, *T*_z_ ⊂ ∆*tℕ*. The time span is chosen according to Nyquist’s sampling theorem to allow a proper reconstruction of a complete 3D period. This is depicted in Fig. 2A.

The *I_z_* sequences are not synchronized in time (Fig. 2A). Let us define a function ϕ : {*I_z_*(*t*)}_*z* ∈ *Z*, *t* ∈ *T_z_*_ → [0,2π) that identifies any image with the corresponding phase of the periodic peristalsis. To reconstruct a full 4D synchronized movie (Fig. 2B), we only need a set *R* = {*t_z_*}_*z* ∈ *Z*_ of reference times *t_z_* ∈ *T_z_* such that the corresponding image of each sequence belongs to the same phase of the peristalsis

$$\phi (I_{m}(t_{m}))=\phi (I_{n}(t_{n}))\forall m,n\in Z$$

This condition alone assures the synchronization of the whole sequence, i.e., ϕ(*I_m_*(*t_m_* + *t*)) = ϕ(*I_n_*(*t_n_* + *t*)). However, relating all lags to a single reference image would not work as images become increasingly difficult to match as ∣*n* − *m*∣ grows. To alleviate this, let us introduce a set *S* = {*s_z_*}_*z* ∈ *Z*_ ⊂ ∆*tℕ* of pairwise relative time lags such that two consecutive slices *I_z_* and *I*_*z*+1_ belong to the same phase, i.e., ϕ(*I_z_*(*t*)) = ϕ(*I*_*z*+1_(*t* + *s*_*z*+1_)) and *s*_*z*+1_ = *t*_*z*+1_ − *t_z_*.

Our strategy consists in combining a relative measure $s_{z+1}^{\text{MI}}$ of the lag with an absolute measure $t_{z+1}^{\text{CI}}$ with respect to the peristaltic motion. The combination is beneficial because we can obtain more accurate estimates of the relative measures (as consecutive slices are more similar) than we can of the absolute measures, but the relative estimates accumulate, and the absolute ones do not. If we were to only use the relative measure, the images would desynchronize over long *z* ranges because their acquisition time varies by ∆*t*; this originally small difference can accumulate over many *z* values to become relevant. Even when less precise, introducing an absolute measure of the phase of the image with respect to the phase of the peristaltic motion ϕ(*I_z_*(*t*)) can feedback into the relative measure and provide stability. In particular, we leverage each measurement according to their relative uncertainty using a Kalman filter (Fig. 2B1)

$$t_{z+1}=(1-K_{z+1})(t_{z}+s_{z+1}^{\text{MI}})+K_{z+1}t_{z+1}^{\text{CI}}$$ (1)

$$K_{z+1}=\frac{\sigma_{z}^{2}}{\sigma_{z}^{2}+\sigma_{\text{CI}}^{2}}$$ (2)

$$\sigma_{z+1}^{2}=(1-K_{z+1})\sigma_{z}^{2}+\sigma_{\text{MI}}^{2}$$ (3)

The gain in Eq. 2 allows us to incorporate both pieces of incoming information in a way that minimizes the variance of the resulting estimate. We now proceed to choose the two measures.

The relative measure is built on mutual information (MI). That is, we find the image in *I*_*z*+1_ that best matches image *I_z_*(*t_z_*) by maximizing their statistical dependency

$$s_{z+1}^{\text{MI}}=\underset{s}{\text{argmax}}\sum_{j\in J}\text{MI}(F(I_{z}(t_{z}+j∆t)),F(I_{z+1}(t_{z}+j∆t-s)))$$ (4)

$$\text{MI}(I_{1},I_{2})≔H(I_{2})-H(I_{2}|I_{1})=\int_{i_{1}\in I_{1}(Ο),i_{2}\in I_{2}(Ο)}p(i_{1},i_{2})\text{log}\frac{p(i_{1},i_{2})}{p(i_{1})p(i_{2})}$$ (5)

where *J* indexes additional images to be matched around the reference (0 ∈ *J*) for better consistency, *p* denotes the probability, and ℱ stands for any filter that may help eliminate possible changes in the image that deviate the signal from perfect periodicity; for example, multiresolution wavelets could alleviate bleaching by cutting off low-pass coefficients. Here, we use three images *J* = {−1, 0, 1} and take the identity for ℱ. We assign an uncertainty of half an exposure time $\sigma_{\text{MI}}^{2}=\text{∆}t/2$ to $s_{z+1}^{\text{MI}}$ according to the accuracy that we expect.

For the absolute measure, we rely on the unnormalized center of intensity

$${\text{CI}}^{+}(I_{z},t)=\int_{x\in Ο}\text{max}\{(x-\mathrm{CT})∙l^{⊥},0\}I_{z}(x,t)$$ (6)

of only half the chip (symmetric stretch) with respect to ***l***^⊥^**∈**ℝ^2^, a unitary vector perpendicular to the long axis ***l*** of the chip, and **CT**, the center of the tissue. We argue that CI^+^ reflects the movement induced by the peristalsis due to the properties of the tissue (see the “Rheological studies” section). Because the induced periodic stretch is typically defined as sin(2π*t*/*T*), then we can fit a general sinusoid on CI^+^(*I_z_*, *t*) for the phase φ and thus find an approximation of the phase map as ϕ = 2π*t*/*T* + φ(CI^+^(*I_z_*)) mod 2π; in other words

$$t_{z}^{\text{CI}}\;\text{mod}\;T=t_{0}-\varphi ({\text{CI}}^{+}(I_{z}))\frac{T}{2\pi }$$ (7)

$t_{z+1}^{\text{CI}}$ is less precise than the relative measurement at $\sigma_{\text{CI}}^{2}=\text{∆}t$, but it is still quite reliable. Other absolute measurements such as OF or edge detectors were tested but discarded because the derivatives involved result in worse error estimates while increasing computational complexity. However, a simple OF measure such as *u*_***l***_^**⊥**^(***x***, *z*, *t*) = −∂_t_*I_z_*(*t*)/(***l***^**⊥**^ ∇_**x**_*I_z_*(*t*)) taken over half the observable tissue eventually becomes more reliable than Eq. 6 in higher-magnification objectives where the tissue covers the whole field of view.

By combining the two measures, we obtain the following uncertainty recursion function

$$\sigma_{z+1}^{2}=\frac{\sigma_{\text{CI}}^{2}\sigma_{z}^{2}}{\sigma_{z}^{2}+\sigma_{\text{CI}}^{2}}+\sigma_{\text{MI}}^{2}=\frac{∆t}{2}(1+\frac{2\sigma_{z}^{2}}{\sigma_{z}^{2}+\Delta t})=\frac{∆t}{2}(3-\frac{2\Delta t}{\sigma_{z}^{2}+\Delta t})$$

which we can actually solve explicitly

$$\begin{array}{c} \sigma_{z+1}^{2}=\frac{∆t}{2}(2-\frac{3}{c4^{z+2}+1}),\\ \sigma_{z+1}^{2}=\frac{∆t}{2}(2-\frac{6}{4^{z+1}+2})\;\text{if}\;\sigma_{0}^{2}=0 \end{array}$$ (8)

Notice that, initially, the filter gain weights the relative measure $s_{z+1}^{\text{MI}}$ more favorably according to the relative proportion of uncertainties, $1-K_{1}=\sigma_{\text{CI}}^{2}/(\sigma_{\text{MI}}^{2}+\sigma_{\text{CI}}^{2})=2/3$. However, as errors accumulate on the estimate *t*_*z*+1_ due to the summation of consecutive lags, the Kalman gain quickly converges to equal proportions independently of ∆*t*: $\underset{z\to \infty }{\text{lim}}K_{z}=1/2$.

The last consideration is the choice of the initial time reference *t*_0_ of the Kalman iteration. Given a sinusoidal peristalsis, we choose *t*_0_ as either the peak, ϕ(*t*_0_) = π/2, because it is easier to recognize, or the phase of maximum change, ϕ(*t*_0_) = π/4, because it makes the MI maxima more prominent. When dealing with multiple colors, each color is synchronized independently and then interpolated to match the exact same time points as those of a chosen reference *T_z_*. We note that, here, the period *T* is imposed by the peristaltic motion but could otherwise be extracted from the dominant Fourier components if it were unknown.

The final 4D reconstruction (Fig. 2B) *I* is the interpolation in *t*, *z* of the map (***x***, *z*, *t*) ↦ *I_z_*(***x***, *t* + *t_z_*) where ***x*** ∈ Ο, *t* ∈ [0, *T*). Note that this reconstruction can be seen as a way to trade time “resolution” for *z* resolution.

#### Manifold projection

The OOCs are not perfectly flat but rather slant significantly in *z* at a rate between 2 and 3 μm every 100 μm along their long axis (Fig. 2B2). As a consequence, part of the tissue is left out of the focal plane (Fig. 2A). As the field of view widens for lower-magnification objectives (notably <20×), the curvature of the tissue becomes important enough to complicate the visualization of the infection process and misdirect automatic quantification. To tackle this issue, we first fit a manifold onto the midsection of the tissue in the 4D reconstruction *I* and then project it back onto the plane.

To find the midsection, we first look for the peak of intensity $z_{c}^{*}(x,t)$ along the *z* axis after presmoothing *I*(*t*) with a Gaussian filter. Although, in this work, we used the wavelet transform to find the maximum in a way that is robust to noise, the original signal appears regular enough for simpler methods to be applicable without relevant loss of accuracy; for example, by directly taking the *z* value of maximum intensity, the results of subsequent processing seem unaffected. On the same processed image, an edge detector is then used to outline the domain of the tissue C ⊂ Ο, whereas the eigenvectors of the image covariance matrix estimate the narrow ***l***^**⊥**^ and long ***l*** axis of the chip. A coordinate transformation *L* : ***x*** → ***x***′***=*** (*x*′, *y*′) from the canonical basis to a basis of ***l***, ***l***^**⊥**^ allows us to take an average $z_{l}^{*}(x\prime ,t)=\int z_{c}^{*}(L^{-1}(x\prime ,y\prime ),t)dy\prime /\int dy\prime$ over the whole length *L*(C)_{*y*^′^}_ of the narrow axis (note that the change of basis is, at most, a combination of a rotation and a translation, and thus, there is no volume change to consider in the integral), along which the position of the tissue does not vary much. Conversely, the variation of the tissue position along the long axis is quite significant, as can be seen by plotting $z_{l}^{*}(x\prime ,t)$ with respect to *x*′ (Fig. 2B3). Combining both observations allows for a map $z*(x,t)=z_{l}^{*}(L{\left(x\right)}_{\left\{x\prime \right\}},t)$ that assigns a *z* position to every point in C.

In turn, this map can be made into a Monge parameterization *p_t_* : ***x*** → (***x***, z*(***x***, *t*)) that captures the 2D manifold *p_t_*(C) = ℳ_t_ embedded in 3D space, as well as the induced metric (see the “Velocimetry and continuum model” section). The parameterization also allows us to project, Π_t_ : ℳ_t_ → ℝ^2^, the tissue back onto a plane in a way that ensures that the flattened tissue, *I*_Π_(*t*), is fully in focus. This can be achieved simply through the transformation $\Pi_{t}=p_{t}^{-1}$ or, more realistically, by preserving the distance according to the arc length along ***l***

$$\Pi_{t}=L^{-1}(\int_{x_{\text{ref}}^{\prime }}^{L{\left(x\right)}_{\left\{x\prime \right\}}}\sqrt{\;1+{\left(\partial_{x\prime }z*(x\prime ,t)\right)}^{2}}dx\prime ,L{\left(x\right)}_{\left\{y\prime \right\}})$$ (9)

which flattens the surface with respect to a reference point $x_{\text{ref}}^{\prime }$ in a way that properly reflects the distances within the tissue (Fig. 2B). In the latter approach, the domains have to be readjusted to fit a possibly longer axis. Either way, the resulting image is then taken as

$$I_{\Pi }(x,t)=I\circ \Pi_{t}^{-1}(x)$$ (10)

#### Movement correction

The peristalsis induced in OOCs also introduces a residual, yet non-negligible (~25 μm), movement in the *z* direction that results in the tissue periodically disappearing (moving out of the focal plane) in each of the *I_z_* sequences (Fig. 2A). To compensate for this *z* movement and separate it from the more interesting dynamics within the manifold ℳ_t_, we devised a time-dependent projection of the reconstructed sequence *I*. Even if we derived it for a fixed image, Eq. 10 constitutes a good basis to extend the projection to account for the time *t*. In particular, because the midsection practically preserves its shape, we propose to fit it with a periodic function to improve time consistency. The periodicity of the peristaltic movement is well reflected on the tissue’s motion due to its rheological properties (see the “Rheological studies” section), and thus, a sinusoid s(*t*) is an excellent fit for the mean of z* (Fig. 2B3). In addition, the movement of *z* does not depend noticeably on ***x***′ at this scale (Fig. 2B3). Therefore, the sequence *I* can be projected (Fig. 2B3) onto the plane as

$$I(\Pi_{t_{0}}^{-1}(x)+s(t)-s(t_{0}))$$ (11)

with respect to a reference *t*_0_ with corresponding manifold ℳ ≔ ℳ_*t*_0__, where ***s***(*t*) = (0,0, s(*t*)) is the fit extended to ℝ^3^ (Fig. 2B3). Similarly, we can also compensate for the *z* movement of the 4D reconstruction:

$$I_{s}(x)≔I(x+s(t)-s(t_{0}))$$ (12)

### Rheological studies

#### Viscoelastic model

Epithelial tissue such as that formed by Caco2 cells on the intestine-chip has been characterized as a viscoelastic solid in the literature. The typical 0D model used to describe its response is known as SLS; it is the simplest model that predicts the phenomena of creep, recovery, and stress relaxation. To reproduce viscoelastic behavior, the SLS uses a particular combination of dashpots and springs, which represent viscosity and elasticity, respectively; they are arranged à la Maxwell as shown in Fig. 3B: parallel [spring, series (spring, dashpot)]. The elastic moduli of the springs are *E*_∞_ and *E*_η_, whereas the viscosity of the dashpot is η. Moreover, we write *E*_0_ = *E*_∞_ + *E*_η_ as the equivalent modulus experienced in an immediate response. We also define the time scale τ_r_ = η/*E*_η_ at which stress relaxes and the retardation time τ_c_ = η*E*_0_/(*E*_∞_*E*_η_) = τ_r_*E*_0_/*E*_∞_, which characterizes the creep of the material (*71*, *72*).

To compute the stress on the tissue, we have to tensorialize this 0D concept into a 2D continuum. In 0D, the equations are built following rules similar to those of electric circuits. If we take σ as the stress and ξ as the strain, the final dynamics are described by

$$\sigma +\tau_{r}\overset{\cdot }{\sigma }=E_{\infty }\xi +E_{\infty }\tau_{c}\overset{\cdot }{\xi }$$ (V1)

This equation is derived by following the parallel-series rules σ = σ_η_ + σ_∞_, ξ = ξ_∞_ = ξ_η_, (where the subscripts ∞ and η denote the spring and Maxwell arms, respectively) as well as the particular dynamics of the damper and springs: $\sigma_{\eta }=E_{\eta }\xi_{\eta ,\text{spring}}=\eta {\overset{\cdot }{\xi }}_{\eta ,\text{damper}},$ ξ_η_ = ξ_η,spring_ + ξ_η,damper_, σ_∞_ = *E*_∞_ξ_∞_.

We tensorialize the equations by following through the 0D derivation but with tensors: ϵ is the strain tensor, ϵ_η_ corresponds to the viscous elements alone, C is the fourth-order stiffness tensor (build-out of the fourth and second rank identities I and **1**), σ is the stress tensor, and ***u*** is the displacement

$$\begin{array}{c} 2\epsilon (u)≔\nabla u+{\left(\nabla u\right)}^{T},\\ C≔\frac{\nu }{\left(1-\nu^{2}\right)}1⨂1+\frac{1}{1+\nu }I \end{array}$$

where we introduce Poisson’s ratio ν according to plane stress conditions. They result in

$$\begin{array}{c} \sigma =C:(E_{0}\epsilon -E_{\eta }\epsilon_{\eta }),\\ {\overset{\cdot }{\epsilon }}_{\eta }=\tau_{r}^{-1}C:(\epsilon -\epsilon_{\eta }) \end{array}$$ (V2)

The time discretization then reads ${\overset{\cdot }{\epsilon }}_{\eta }^{t+1}=(\epsilon_{\eta }^{t+1}-\epsilon_{\eta }^{t})/\Delta t$ as a backward Euler scheme with Δ*t* the time. See (*73*) for reference.

#### Experiments

There are several experiments that material scientists harness to measure the constants. The most pertinent here are those of relaxation, constant rates, and creep. Relaxation experiments track the exponential decrease (−*t*/τ_r_) of stress from the instant response (*E*_0_) to the limit response (*E*_∞_) in the face of a strain step (ξ_0_)

$$\sigma (t)=\xi_{0}E_{\infty }(1+(E_{\eta }/E_{\infty })e^{-t/\tau_{r}})$$

The constant rate experiments study the response of the stress to a linear increase in strain and vice versa (fig. S1C). Creep experiments look at the (−*t*/τ_c_) growth of strain from the instantaneous to the limit response in the face of a stress step (ξ_0_), whereas creep recovery experiments look at the decrease (−*t*/τ_c_) in strain to zero once the stress is removed (Fig. 3B and fig. S1A). Respectively

$$\begin{array}{c} \xi (t)=(\sigma_{0}/E_{\infty })(1-(\frac{E_{\eta }}{E_{0}})e^{-\frac{t}{\tau_{c}}}\;),t<t_{0}\\ \xi (t)=(\sigma_{0}E_{\eta }/(E_{\infty }E_{0}))(e^{-\frac{t-t_{0}}{\tau_{c}}}-e^{-\frac{t}{\tau_{c}}}\;),t\geq t_{0} \end{array}$$

with *t*_0_ the moment when stress drops. Neither the relaxation nor the constant rate options are applicable to our pump: We can only control the stress, not strain, and we cannot follow the strain properly when it moves quickly because the images go out of plane. Because the pressure pump cannot recreate a quick enough “instant” rise in stress to study the creeping behavior (fig. S1A), we instead focus on the pressure drop to assess creep recovery and extract τ_c_. To measure *E*_∞_, we can conduct the classic experiment for Young’s modulus if we wait for the material to reach equilibrium at each pressure step (i.e., *t* ≫ τ_c_). Poisson’s ratio ν can be extracted by comparing the strain in perpendicular directions. Last, the dephasing between stress and strain under a sinusoid input can also help measure some of the constants (see the “Complex analysis” section). We now go over each of these experiments individually.

##### Creep recovery

To measure the retardation time, we apply a boxcar pressure (0 to −700 mbar to 0 mbar again; see fig. S1A) with the pump, which expands the chip crosswise. When the tissue is expanded, it goes out of plane only to come back when the pressure drops. We stay on the initial plane imaging at a high rate to capture the last part of the creep recovery (fig. S1A). To extract τ_c_, the strain of the tissue ${\bar{\epsilon }}_{y^{\prime },y^{\prime }}(t)$ in the ***l***^⊥^ direction is measured and fitted with an exponential function via a linear regression of its logarithm (fig. S1A, inset) with respect to time (i.e., inverting from the slope −1/τ_c_). Note that we take the average ${\bar{\epsilon }}_{y^{\prime },y^{\prime }}(t)$ over the tissue of ϵ_*y*^′^,*y*^′^_(***x***′, *t*) because the latter is constant. This is because ***u***_*y*′_ is a linear function of *y*′ (see fig. S1A and the “Complex analysis” section). Here, ϵ(***u***) is computed by first measuring ***u***(***x***, *t*) in either of two ways: one, by using OF on the E-cadherin–labeled tissue images to measure the displacement between pairwise frames ***u***_***t***_(***x***) (see the “Velocimetry and continuum model” section and Fig. 3C and fig. S1A) and then accumulating the displacements as $u(x,t_{c})=\sum_{t=t_{c}}^{t=t_{f}}u_{t}$, where *t*_f_ is the final time when the tissue is already at rest; and two, the holes in the PDMS membrane can be also used as reference; they are detected automatically as spots (spot detector) using multiresolution analysis with the stationary wavelet transform (same way as in quantitative measures) and then tracked using MH tracking to compensate the nearest neighbors for any possible undetected spot in an image. Both approaches yield the same conclusions.

##### Young’s modulus

To measure *E*_∞_, we apply a linear range of pressures *P* (from 0 to −700 mbar; see fig. S1B). At each pressure step, we wait for equilibrium and take a 3D acquisition. OF (see the “Velocimetry and continuum model” section) is then used to extract the displacement between acquisitions and build ***u*** by accumulation. Last, a linear fit of the strain ${\bar{\epsilon }}_{y^{\prime },y^{\prime }}$ as a function of *P* reveals *E*_∞_ in its slope (see fig. S1B).

##### Poisson’s ratio

To measure ν, we take profit of the previous experiments to compute ν = **−**ϵ_*y*^′^,*y*^′^_/ϵ_*x*^′^,*x*^′^_ [see (*74*) for comparison to an alternative method].

#### Complex analysis

To analyze the expected response of the tissue to a sinusoidal input (*75*), we feed the system a complex function ∝ *e*^*i*ω*t*^. The conclusion will be that a sinusoidal strain gives rise to a phase-shifted (by δ) sinusoidal stress of the same frequency and vice versa. To prove this, take a complex strain ·ξ* = ξ_0_*e*^*i*ω*t*^ and thus a rate $\overset{\cdot }{\xi *}=i\omega \xi *$; use a proxy σ* = σ_0_*e*^*i*ω*t*^ with unknown σ_0_ as tentative solution and plug it in Eq. V1

$$\sigma_{0}=\xi_{0}E_{\infty }\frac{1+i\omega \tau_{c}}{1+i\omega \tau_{r}}$$

We can then define a complex modulus E* such that σ* = E*ξ*

$$E*(\omega )=E_{\infty }+E_{\eta }\frac{i\omega \tau_{r}}{i\omega \tau_{r}+1}$$ (V3)

Therefore, the sinusoidal response has a phase shift of δ = arg (E*) and an amplitude modulation of ∣E*∣. The maximum phase is then achieved at ωτ_r_ = 1 with a value of approximately *E*_η_/(2*E*_∞_ + *E*_η_). Equation V3 can be used to deduce any remaining constant (namely, *E*_η_) out of the rest (measured above) from a measure of the phase lag between the stress (pump) and the strain (images).

#### Decoupling tissue and chip

While the flexibility of the experimental setting is very limited (pump, chip, imaging, etc.), in the “Experiments” section, we have devised work-arounds to measure the constants characterizing the chip. In this way, we also confirmed that the system is viscoelastic. However, it is hard to decouple the effect of the tissue and the chip membrane itself, which is made of PDMS, a viscoelastic material too (albeit with a lesser viscous component than the tissue).

To explore the contribution of both the tissue and the chip to creep recovery, we used τ_c_ to measure the perceived viscoelasticity of the chip (see the “Experiments” section) before and after adding trypsin. The trypsinization process removes the tissue from the chip, in live, by detaching the cells and flushing them and thus lets us take before and after measurements at the same spots consistently. Three measurements were taken at each of the different spots: one at an initial time (0 hour); another after 2 hours, right before adding trypsin; and a third at 3 hours, an hour after trypsinization. The resulting values of τ_c_ did not change over the first 2 hours (1.02 ± 0.03 s at 0 hour, 1.00 ± 0.04 s at 2 hours, SD) only to default to the range of PDMS values found in the literature (*45*, *76*) after the trypsinization process (0.68 ± 0.02 s at 2 + 1 hours, *P* = 1 × 10^−6^, *n* = 4). The value of τ_c_ reported in (*45*) for PDMS varies according to polymer cross-linking, which is regulated by the concentration of linker enzymes during polymerization; the rest of constants do not seem to be indicative in this respect. Therefore, it appears that τ_c_ reflects how well meshed a material is and thus could serve as a marker for the state of the tissue. We have seen that τ_c_ captures the progressive destruction of the tissue by amoebic parasites (Fig. 6A) in a more reliable way than the connectivity measure. This is convenient because the retardation time is easier to measure than the relaxation time in this setting.

Conversely, we could not differentiate the Young’s modulus corresponding to the tissue from that of the PDMS, *E*_∞_ = *E*_∞,PDMS_ + *E*_∞,tissue_, because the latter is expected to be an order of magnitude higher. The values before (967 ± 16 kPa, SD) and after (950 ± 13 kPa) trypsinization were not significantly different (*P* = 0.35, *n* = 4). To notice a difference upon trypsinization of the chip, we would require a resolution higher than is now available to us, probably that of a 63× objective. As a reference, the values expected for the tissue’s elasticity modulus range around 10 to 1 kPa [e.g., 20 kPa in (*43*) or 0.64 kPa in (*44*)].

Fortunately, the remaining constants that we could not measure accurately enough are only relevant to the magnitude of the stress (Eq. V2), but not to its spatial distribution (Eq. V2) and therefore neither to the correlation analysis in the “Hypothesis Testing” section of statistical tests. The lag between stress and strain does not influence the correlation analysis either because the latter is performed on the time integral. In other words, the results are true up to a constant.

#### Related conclusions

In conclusion, despite our limited capabilities, we were able to confirm the viscoelasticity of the tissue. Our analysis reveals that creep recovery of the tissue can be measured effectively with the pump through τ_c_ and that it reflects how well cross-linked is the material, serving as a probe for tissue destruction, and that this is independent of the effect of the PDMS in the surrounding chip as tested with trypsin. Conversely, Young’s modulus would require additional magnification power to be resolved in a way that decouples tissue and chip. However, the complex analysis suggests that the constants are practically irrelevant to the statistical correlation with infection foci because everything is defined up to a constant and a phase lag.

### Velocimetry and continuum model

#### Optical flow

OF works under the assumption of brightness conservation to estimate the displacement ***u*** between a pair of images. The linearized equation describing OF on the plane reads (*46*)

$$\partial_{t}I+<u,\nabla^{e}I{>}_{e}=0$$ (O1)

with the standard Euclidean inner product (e). Let us reformulate this concept on the tissue manifold ℳ. We need to compensate for the *z* movement using *I*_***s***_, force the image gradient to stay on the manifold, account for the different inner product to correctly represent the angles between vectors, and define the in-manifold deformation or displacement. Let us denote the tangent bundle of ℳ as *T*ℳ [e.g., (*42*, *77*)]. We are looking for a vector field ***u*** : ℳ → *T*ℳ that describes the deformation within the manifold. The inner product on the manifold ***<*** · , · ***>***_ℳ_**:***T*ℳ × *T*ℳ → ℝ is known as the first fundamental form and can be represented by the matrix

$$\left(\begin{array}{cc} 1+{\left(\partial_{x\prime }z*\right)}^{2} & 0\\ 0 & 1 \end{array}\right)$$

on the basis of tangent vectors ***e***_*x*′_ = (1,0, ∂_*x*^′^_z*) and ***e***_*y*′_ = (1,0,0), where we reparameterized ***x*** into ***x***′ because ∂_*y*′_z* = 0. Therefore, we have $a=\sqrt{1+{\left(\partial_{x\prime }z*\right)}^{2}}$ for the area of a surface element (*d*ℳ = *a dx*’*dy*’) and ***n*** = (−∂_*x*^′^_z*,0,1)/*a* for the local unit vector normal to ℳ. On the other hand, the in-manifold or tangential derivative can be written as the Euclidean projection of the intensity gradient from the ambient space onto the tangent space of the manifold, i.e., ∇^ℳ^*I*_***s***_ = ∇^e^*I*_***s***_ − ***<*** ∇^e^*I*_***s***_, ***n>***_e_***n***, ∇^ℳ^*I*_***s***_** ∈ ***T*ℳ. With these three ingredients, we can rewrite Eq. O1 into

$$\partial_{t}I_{s}+<u,\nabla^{M}I_{s}{>}_{M}=0$$ (O2)

This problem can be solved by embedding the equation in a variational framework. In this case, by integrating over a full period (represented by the set *T*_0_ and considering ***u*** over time too), we do not only enforce spatial consistency but also temporal

$$J_{\text{OFM}}≔\sum_{ti\in T_{0}}\int_{M}{\left(<T_{i}u,\nabla^{M}I_{s}(ti+\Delta t){>}_{M}+I_{s}(ti+\Delta t)-I_{s}(ti)\right)}^{2}dM$$ (O3)

where $T_{i}u≔\int_{t_{i}}^{t_{i+1}}u$ stands for the observations because ***u*** is the total displacement field present in the viscoelastic equations. Notice that the magnitude of ***u*** grows progressively, and thus, there is no need for a multiresolution scheme. The problem is then

$$\begin{array}{c} \underset{u}{\text{argmin}}\;J_{\text{OFM}}+J_{\text{REG}}\\ J_{\text{REG}}=\alpha \sum_{t\in T_{0}}\int_{M}\nabla^{M}u:\nabla^{M}u+\partial_{t}u\cdot \partial_{t}u\;dM \end{array}$$ (O4)

where α ∈ ℝ_>0_ is a weighting parameter and ∇^ℳ^***u*** is the covariant derivative, which extends the notion of tangential derivative to vector fields by accounting not only for the change in the function itself but also for the change in the reference axis, ***e***_*x*^′^_(***x***′), ***e***_*y*′_(***x***′), as the manifold curves. It reads $\nabla_{e_{i}}^{M}u=(\partial_{e_{i}}u^{k}+\Gamma_{i\;j}^{k}u^{j})e_{k}$ (Einstein summation) because ***u*** is a displacement vector and thus contravariant, with $\Gamma_{i\;j}^{k}$ the Christoffel symbols. Problem Eq. O4 is solved by first meshing the manifold and then using the finite element method (FEM) (*78*–*80*) on the variations resulting from Eq. O4.

OF is convenient in its variational formulation because it combines well with the weak form of the continuum mechanics below to enable a unifying FEM framework. Moreover, much like that reported in the literature, we have found OF to outperform other particle image velocimetry (PIV) methods in similar contexts. That said, it is likely that the results of other PIV approaches would remain sufficiently accurate to the analysis of the images and allow for the same conclusions.

#### Stress

Once ***u*** is recovered (Fig. 3C), the stress (Fig. 3D) can be computed directly through Eq. V2 but with the derivatives [e.g., (*81*, *82*)] adapted to those on the manifold

$$\begin{array}{c} 2\epsilon (u)≔\nabla^{M}u+{\left(\nabla^{M}u\right)}^{T},\\ C:\epsilon =\frac{\nu }{\left(1-\nu^{2}\right)}\text{tr}(\epsilon )1+\frac{1}{1+\nu }(\epsilon ) \end{array}$$

However, notice that we do not know the initial condition of the stress. If the starting image could be chosen, then the initial stress could be potentially set to zero. Alternatively, we could perform multiple passes to reach a steady-state solution starting from a zero guess, but the temporal discretization is likely to create a fake time lag. Therefore, we formulate a partial differential equation(s) (PDE)-constrained problem

$$\underset{g,\sigma (t_{0})\;}{\text{argmin}}\;J_{\text{OFM}}+J_{\text{REG}}\;\text{subject to d}=0$$ (O5)

$$\begin{array}{c} d=\nabla^{M}\cdot \sigma \;\text{in}\;M,\\ d=u-g\;\text{on}\;\partial M, \end{array}$$

$$\begin{array}{cc} J_{\text{REG}} & =\alpha \sum_{t\in T_{0}}\int_{\partial M}g\cdot g+\partial_{t}g\cdot \partial_{t}g\\ & +\beta \int_{M}\sigma (t_{0}):\sigma (t_{0})\\ & +\gamma \int_{M}(\sigma (t_{0}+T)-\sigma (t_{0})):(\sigma (t_{0}+T)-\sigma (t_{0})) \end{array}$$

the controls of which are the boundary conditions ***g*** and the initial stress σ(*t*_0_) and where β, γ ∈ ℝ_>0_. The problem is solved using the adjoint method to compute the functional derivatives (with a check-pointing scheme) and the FEM [see (*83*, *84*) for context], where the elastic equations are transformed into their corresponding weak form by integration over the manifold norm. Here, the weak equations also need to be integrated over time to form the Lagrangian together with the data and regularization functionals. Further integration by parts prompts the appearance of an integral evaluated at the final time that can be eliminated by setting a terminal condition on the test function and later on the adjoint. The derivative with respect to ***g*** (for example) depends on time because ***g*** does and because the adjoint variables do, i.e., the full-time history of the adjoint variables is required. While the regularization terms here are written in *L*^2^, we have found that the reconstructions are more stable when using *H*^1^. The final results of solving Eq. O5 are estimates of the ***u*** and the stress σ at once. In summary, we have decomposed the total movement into that of *z* (see the “Movement correction” section) and that in-manifold (see this subsection); together, they explain the full movement of the tissue.

### Hypothesis testing

To compare the different infection markers (e.g., penetration or TSAR activation) with a measure of the local stress, we choose the average stress map over a full peristaltic cycle

$$\sigma_{T}(x)≔\frac{1}{T}\int_{t_{0}}^{t_{0}+T}\sigma :\sigma \;\mathrm{dt}$$

The correlation between this measure and the markers can then be computed in two ways: comparing image to image (e.g., stress versus penetration values) or image to weighted positions (e.g., stress versus TSAR activations). We use the term images because this type of correlations have been studied before in biological settings, albeit under slightly different assumptions (*47*, *48*, *85*). However, the adaptation to our problem only involves replacing the null hypothesis for the distribution measured without stress (i.e., WOP) and adding weights (in this case, the measures).

## Notes

View/request a protocol for this paper from *Bio-protocol*.
