# Supplementary material for: 4D live imaging and computational modeling of a functional gut-on-a-chip evaluate how peristalsis facilitates enteric pathogen invasion
Source: Sci Adv. 2022 Oct 21;8(42):eabo5767. doi: 10.1126/sciadv.abo5767 (PMC9586479; doi:10.1126/sciadv.abo5767)
Supplement: Supplementary file 1 — Figs. S1 and S2 [file sciadv.abo5767_sm.pdf]

Science Advances
AAAS

Supplementary Materials for

4D live imaging and computational modeling of a functional gut-on-a-chip evaluate how peristalsis facilitates enteric pathogen invasion

Aleix Boquet-Pujadas *et al.*

Corresponding author: Aleix Boquet-Pujadas, aleix.boquetipujadas@epfl.ch;
Jean-Christophe Olivo-Marin, jcolivo@pasteur.fr; Nathalie Sauvonnet, nathalie.sauvonnet@pasteur.fr;
Elisabeth Labruyère, elisabeth.labruyere@pasteur.fr

Sci. Adv. **8**, eabo5767 (2022)
DOI: 10.1126/sciadv.abo5767

The PDF file includes:

Figs. S1 and S2
Legends for movies S1 to S8

Other Supplementary Material for this manuscript includes the following:

Movies S1 to S8

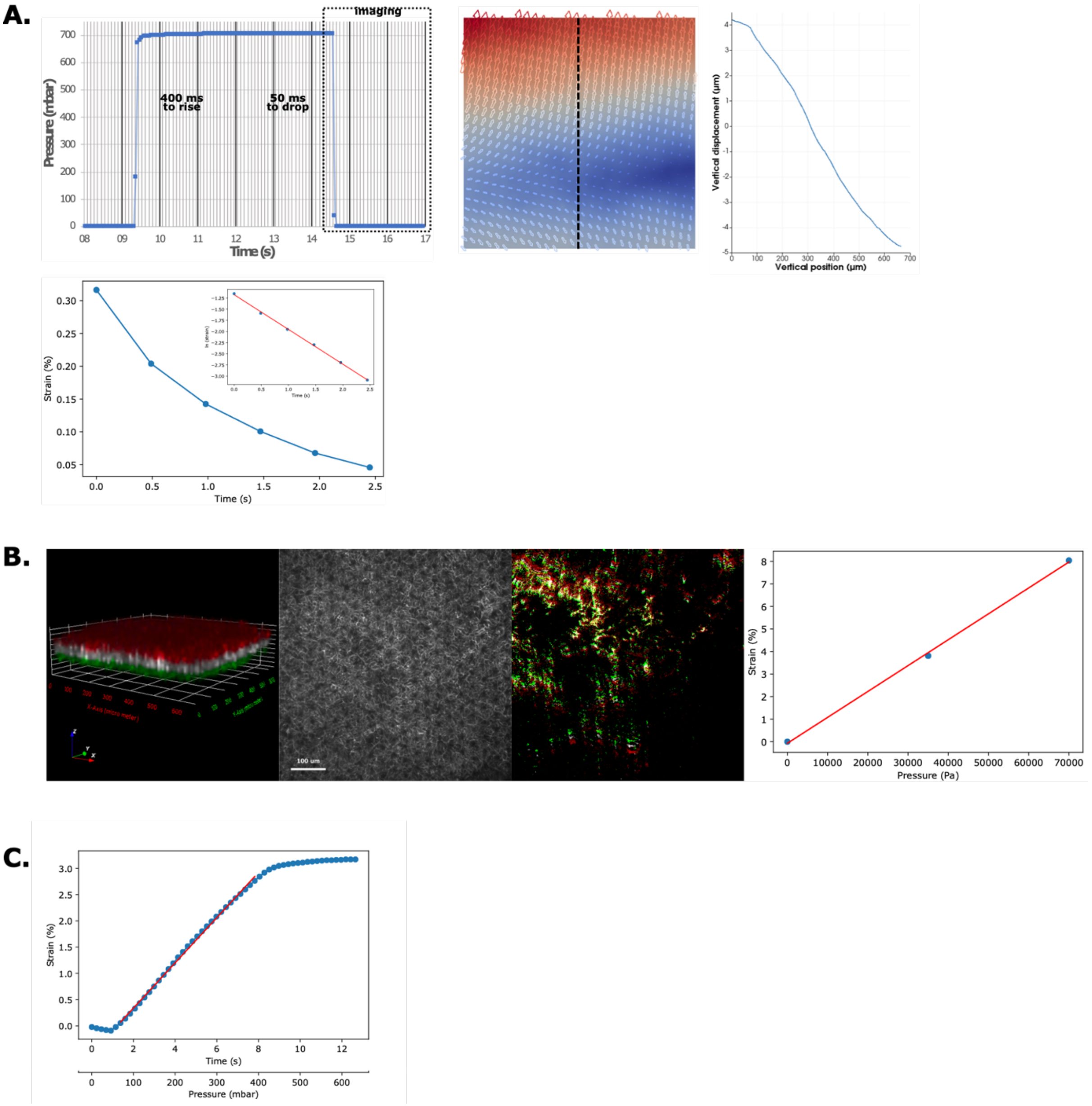

Fig. S1.

Viscoelasticity experiments with the pressure pump. A.i) Pressure applied by the pump to the OOC in the y direction. We use the pressure drop (as it is quicker than the pressure rise) to image and measure the retardation time. **A.ii)** Displacement map of the tissue (blue low, red high) at a single time point while it relaxes during retardation. **A.iii)** Displacement profile across the dashed black line in (A.ii). The profile is almost linear, meaning the slope is constant and can thus characterize the strain (derivative of displacement in y) of the tissue at each time point. **A.iv)** Strain of the tissue at each time point as measured in (A.ii-iii) show an exponential, therefore the slope of the linear fit of the logarithm (inset) reveals the characteristic (retardation) time.

B.i) Three colors represent the tissue in 3D at three different pressures (green 0kPa, white 35kPa, red 70kPa) after relaxation, showing how it moves in z. **B.ii)** 20x zoom-in of the tissue. **B.iii)** Three colors represent the tissue in a 2D confocal slice at the three different

pressures after relaxation, showing how it stretches in x-y. **B.iv)** By extracting the strain in the same way as (A.ii-iii) we find a linear relation between stress and strain.
C) Response of the tissue strain to a growing linear pressure by following the changing focal plane with a specially programmed microscope.

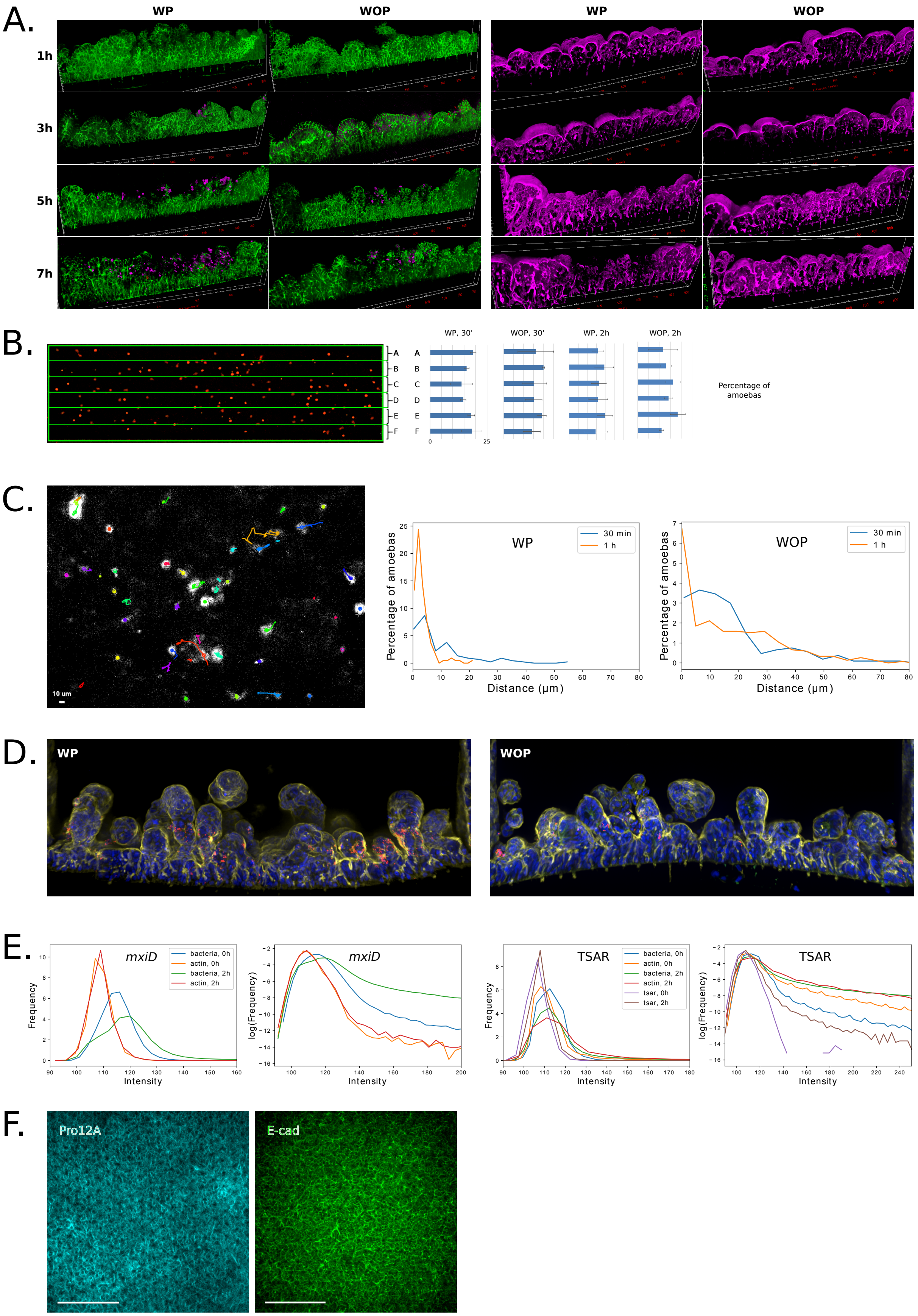
A.
WP
WOP
WP
WOP
1h
3h
5h
7h
B.
A
B
C
D
E
F
WP, 30'
WOP, 30'
WP, 2h
WOP, 2h
0
25
Percentage of amoebas
C.
10 um
WP
WOP
30 min
1 h
Percentage of amoebas
Distance (μm)
D.
WP
WOP
E.
mxiD
TSAR
bacteria, 0h
actin, 0h
bacteria, 2h
actin, 2h
tsar, 0h
tsar, 2h
Frequency
log(Frequency)
Intensity
F.
Pro12A
E-cad

Figure S2.

Fixed and quantitative studies of pathogen invasion of OOCs. A) Destruction of the tissue over time by *E. histolytica*. 3D perspectives of fixed tissue slices imaged as z-stacks in a confocal microscope. Tissue were fixed at 1h, 3h, 5h and 7h of infection, WP and WOP (8 chips total). The channel corresponding to amoebae is not present. In the images of two first columns are visible the E-cadherin (green) and dead cells (violet), and in the images of the two last columns only the actin (violet) is visible. **B)** Example images of the repartition of amoebae (red) on tissue with the image divided in different zones. On the right, the percentage of amoebae found on each zone is shown for WP and WOP at 30min and 2h. No difference is statistically significant**. C)** Example x-y view of amoebae (white) trajectories (multi-colors) during the first 30 minutes post-challenge. On the right, WP and WOP histograms summarizing the statistical distribution of the length of amoebae trajectories at 30min and 2h. **D)** 2D projection of fixed tissue slices imaged as z-stacks in a confocal microscope at 3h post-challenge with *S. flexneris*. DAPI in blue, actin in yellow and bacteria in red. E) Intensity histograms (both normal and logarithmic) of the actin, bacterial and TSAR activation signals to compare the *mxiD* strain (which does not recruit actin as shown by the fact that the histogram tails do not get heavier) with the TSAR strain, where the tails of all the three signals get heavier with time. F) Comparison between membrane labelling with the Pro12A probe (cyan) of Caco2 cells and constitutive expression of E-cadherin-GFP in Caco2 cells. Scale bars are 20µm-long.

Movie S1.
Confocal slice of intestine-chip 10x. Movie of a confocal slice of an intestine-chip matured with Caco2 E-cadherin-GFP using a 10x objective. The movie is in real time over a full peristaltic cycle for brevity (cf. Figure 2 for scale).

Movie S2.
Confocal slice of intestine-chip 20x. Movie of a confocal slice of an intestine-chip matured with Caco2 E-cadherin-GFP using a 20x objective. The movie is in real time over a full peristaltic cycle for brevity.

Movie S3.
Projection of 4D reconstruction onto manifold 10x. Projection onto the tissue manifold of the 4D sequence reconstructed from Movie 1.

Movie S4.
Projection of 4D reconstruction onto manifold 20x. Projection onto the tissue manifold of the 4D sequence reconstructed from Movie 2.

Movie S5.
On-manifold displacement of the tissue with underlying *Shigella flexneri*. Displacement within the tissue manifold (arrows: blue low to red high) with tissue in greyscale and some bacteria in violet (contrast enhanced). The movie is in real time over a full peristaltic cycle.

Movie S6.
On-manifold stress of the tissue with underlying *Shigella flexneri*. Stress within the tissue manifold corresponding to Movie 6.

Movie S7.
On-manifold displacement of the tissue with underlying *Entamoeba histolytica*. Displacement within the tissue manifold (arrows: blue low to red high) with tissue in greyscale and some amoebae in violet (contrast enhanced). The movie is in real time over a full peristaltic cycle.

Movie S8.
On-manifold stress of the tissue with underlying *Entamoeba histolytica*. Stress within the tissue manifold corresponding to Movie 7.
